# Lipid-driven membrane remodeling engages a lysosome-dependent adaptive repair program during retinal aging

**DOI:** 10.1186/s13024-026-00990-w

**Published:** 2026-08-29

**Authors:** Emily Tom, Fangyuan Gao, Carolina N. Franco, Adrian Wong, Nathan Kemmerer, Zichen Wang, Amit Jairaman, Qianlan Xu, Yinyin Zhuang, Samuel W. Du, Grazyna Palczewska, Krzysztof Palczewski, Itay Budin, Shivashankar Othy, Xiaoyu Shi, Vera L. Bonilha, Johannes Schoeneberg, Karl J. Wahlin, Lauren V. Albrecht, Dorota Skowronska-Krawczyk

**Affiliations:** 1 Department of Physiology and Biophysics, School of Medicine, University of California, Irvine, Irvine, CA USA; 2 Gavin Herbert Eye Institute – Brunson Center for Translational Vision Research, Department of Ophthalmology and Visual Sciences, University of California, Irvine, Irvine, CA USA; 3 Department of Pharmaceutical Sciences, School of Pharmacy and Pharmaceutical Sciences, University of California, Irvine, Irvine, CA USA; 4 Department of Chemistry and Biochemistry, University of California San Diego, La Jolla, CA USA; 5 Shiley Eye Institute, University of California, San Diego, La Jolla, CA USA; 6 Department of Pharmacology, University of California San Diego, La Jolla, CA USA; 7 Department of Developmental and Cell Biology, University of California, Irvine, Irvine, CA USA; 8 Department of Molecular Biology and Biochemistry, University of California, Irvine, Irvine, CA USA; 9 Department of Chemistry, University of California, Irvine, Irvine, CA USA; 10 Department of Biomedical Engineering, University of California, Irvine, Irvine, CA USA; 11 Department of Ophthalmic Research, Cleveland Clinic, Cleveland, OH USA; 12 Department of Molecular Medicine, Cleveland Clinic Lerner College of Medicine, School of Medicine, Case Western Reserve University, Cleveland, OH USA

**Keywords:** Membrane remodeling, Aging, Neurodegeneration, Lysosomal exocytosis, Age-related macular degeneration (AMD)

## Abstract

Age-associated remodeling of membrane lipid composition has been implicated in cellular dysfunction, yet the mechanisms linking lipid changes to membrane integrity and disease remain poorly defined. In the retinal pigment epithelium (RPE), lipid dysregulation is strongly associated with aging and age-related macular degeneration (AMD), a neurodegenerative disease of the central nervous system, but the causal pathways remain unclear. Here, we identify reduced activity of the lipid elongase ELOVL2 as a central driver of age-dependent membrane remodeling. Loss of ELOVL2-dependent polyunsaturated fatty acid (PUFA) elongation shifts plasma membrane lipid composition, leading to altered membrane biophysical properties and compromised membrane integrity. In response to this stress, RPE cells do not undergo apoptosis but instead activate a lysosome-dependent plasma membrane repair program that preserves barrier function under metabolic challenge. However, this adaptive response drives spatially polarized lysosomal exocytosis, promoting extracellular remodeling and accumulation of sub-RPE deposits associated with aging and AMD. Restoration of ELOVL2-derived lipid products reverses membrane abnormalities and suppresses lysosome-mediated remodeling phenotypes, demonstrating direct metabolic control of membrane homeostasis. Together, these findings define an ELOVL2-dependent lipid–lysosome axis that links PUFA elongation to plasma membrane integrity and reveals how compensatory repair mechanisms can contribute to tissue remodeling and disease progression in aging epithelia.

**Supplementary information:**

The online version contains supplementary material available at https://doi.org/10.1186/s13024-026-00990-w.

## Introduction

Aging is associated with widespread remodeling of cellular membranes, yet how these changes manifest at the tissue level and contribute to functional decline and neurodegeneration within the central nervous system remains incompletely understood. The retinal pigment epithelium (RPE) provides a powerful model to study these processes, as it forms a highly specialized, long-lived monolayer that maintains outer blood-retina barrier integrity while supporting photoreceptor health and visual function through active membrane trafficking, nutrient exchange, and daily phagocytosis of photoreceptor outer segments [1]. As a neuroepithelial tissue and integral component of the central nervous system, the retina is particularly vulnerable to age-associated metabolic and membrane dysfunction. These demands place exceptional reliance on the stability and adaptability of the plasma membrane. With age, the RPE undergoes progressive metabolic and structural decline, characterized by altered lipid handling, impaired lysosomal clearance, and the accumulation of extracellular debris – hallmarks observed in both physiological aging and age-related macular degeneration (AMD) [2, 3], main cause of vision loss and blindness in older adults. Despite this well-defined phenotype, how age-associated metabolic changes are integrated at the level of membrane organization, tissue remodeling and neurodegenerative progression remains poorly defined.

Cellular membranes are dynamic structures whose lipid composition and biophysical properties shape essential cellular functions, including vesicular trafficking, signaling, and stress responses [4, 5]. These properties are especially critical in post-mitotic RPE cells, which must preserve membrane integrity over decades while exposed to chronic mechanical, metabolic, and oxidative stress [6]. Accordingly, the RPE represents a uniquely sensitive system for studying how disruptions in lipid metabolism impact membrane homeostasis. Among membrane lipids, polyunsaturated fatty acids (PUFAs), such as docosahexaenoic acid (DHA, 22:6n-3), are key determinants of membrane flexibility and function, supporting curvature, diffusion, and membrane dynamics required for RPE physiology [7]. The enzyme ELOVL2 (Elongation of Very Long-Chain Fatty Acids-Like 2) is a central regulator of PUFA elongation, controlling the biosynthesis of long-chain and very-long-chain PUFAs, including DHA. Notably, ELOVL2 has emerged as one of the most robust molecular biomarkers of aging, with progressive promoter methylation tightly correlated with chronological age [8–10]. In the retina, reduced ELOVL2 expression is associated with altered lipid composition and early functional decline [11, 12], and restoration of its metabolic products improves visual function in aged mice [12]. More broadly, aging is accompanied by coordinated shifts in cellular lipid composition, including changes in PUFA abundance, sphingolipid content, and phospholipid class balance, which alter membrane biophysical properties [13–15] and perturb essential membrane-dependent cellular processes [16], though a mechanistic understanding remains enigmatic.

Given the central role of PUFA metabolism in membrane stability, we hypothesized that reduced ELOVL2 activity represents a key metabolic node linking age-associated lipid remodeling to RPE dysfunction. To address this, we leveraged a multi-scale experimental framework combining human aging RPE datasets, ELOVL2-deficient mouse models, and in vitro RPE systems, including induced pluripotent stem cell–derived RPE (iPSC-RPE). Across these complementary models, we identify a conserved signature of membrane remodeling associated with aging and ELOVL2 loss, characterized by altered plasma membrane lipid composition and activation of lysosome-dependent adaptive responses. These findings establish the RPE as an integrative model for studying how age-dependent metabolic shifts reshape membrane organization and drive tissue remodeling, providing insight into how lipid dysregulation contributes to functional decline in aging epithelia.

## Results

### Transcriptomic profiling reveals age-related membrane remodeling in the human and mouse RPE

**Fig. 1:**
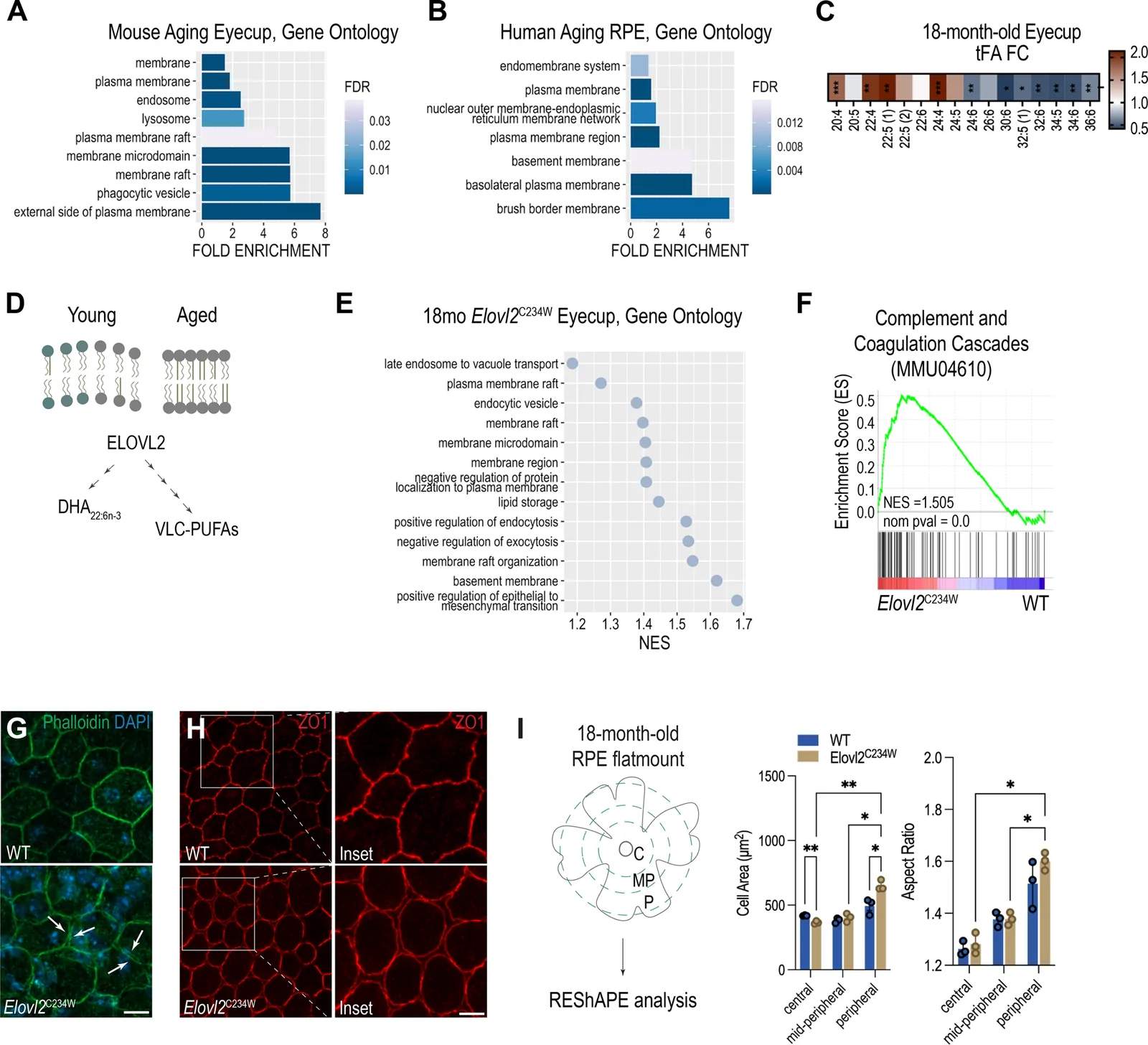
ELOVL2 depletion recapitulates age-associated membrane remodeling during aging in the RPE. (**A**) Cellular Component overrepresentation analysis of differentially expressed genes (DEGs) from 18-month-old wild-type mouse eyecups. (**B**) Cellular Component overrepresentation analysis of significantly correlated genes (SCGs) from human aging RPE [17]. (**C**) Heatmap of total fatty acid changes in eyecups from 18-month-old wild-type mice relative to 3-month-old wild-type mice (*n* = 5 per group). (**D**) Schematic illustration of the role of ELOVL2 in the synthesis of LC- and VLC-PUFAs and the effect of its loss on membrane lipid composition during aging. (**E**) Gene set enrichment analysis (GSEA) of eyecups from 18-month-old *Elovl2*^C234W^ mutant mice using the Gene Ontology Cellular Component ontology. NES, Normalized Enrichment Score. (**F**) GSEA enrichment plot for the “complement and coagulation cascades” term (KEGG mmu04610) in *Elovl2*^C234W^ eyecups. (**G, H**) Representative confocal images of RPE flat mounts from 18-month-old wild-type and *Elovl2*^C234W^ mice stained with phalloidin (green) and DAPI (blue) (**G**) and ZO-1 (red) (**H**). White arrows indicate RPE stress fibers. Scale bars, 20 μm; inset, 10 μm. (**I**) *Left:* schematic of central, midperipheral, and peripheral RPE regions used for morphometric analysis. *Right:* Quantitative morphometric analysis of RPE cell area and aspect ratio using ReSHAPE (*n* = 3 animals per group). Data are mean ± SEM. Statistical significance was determined by two-way ANOVA with Geisser–Greenhouse correction. *p < 0.05, **p < 0.01, ***p < 0.001, ****p < 0.0001. Bulk RNA sequencing data from mouse eyecups have been deposited in GEO repository under accession number GSE343357 (**A, D, E**). Lipidomics data from mouse eyecups have been deposited in dryad (DOI https://doi.org/10.5061/dryad.34tmpg51p) (**B**). Human aging RPE RNA sequencing data was obtained from GEO repository under accession number GSE159435 (**C**)

Our study aimed to explore the impact of aging on the membrane-associated processes in RPE. To address this, we performed bulk RNA sequencing on RPE–choroid eyecups from young (3-month-old) and aged (18-month-old) wild-type mice. Differential expression and pathway enrichment analyses showed significant enrichment of genes associated with membrane components, including plasma membrane, membrane microdomain, lysosome, and endosome categories in aged mouse eyecups (Fig. 1A). To determine whether similar membrane-associated transcriptional signatures occur in humans, we next analyzed transcriptomic data from a previously published study of healthy aging human RPE donors aged 31 to 93 years (GSE159435) [17]. We extracted significantly correlated genes (SCGs) exhibiting a significant positive or negative correlation with age (Benjamini–Hochberg adjusted *p* < 0.05 and |βage| >0.025). Overrepresentation analysis of downregulated SCGs using the PANTHER Gene Ontology (GO) “Cellular Component” category [18] revealed significant enrichment of membrane-associated structures, including the endomembrane system, plasma membrane, and brush border membrane (Fig. 1B). Together, these data demonstrate that aging-associated total membrane remodeling signatures are conserved across mouse and human RPE.

Given the strong enrichment of membrane-associated pathways in aging, we next investigated whether altered lipid metabolism may underlie these changes. Total fatty acid profiling of all cellular lipids from young (3-month-old) and aged (18-month-old) wild-type eyecups revealed selective remodeling of PUFA species, with significant depletion of long- and very-long-chain PUFAs in aged tissue, consistent with reduced activity of PUFA elongation pathways downstream of ELOVL2 (Figs. 1C, D, S1A). These age-associated changes in cellular PUFA composition suggested that altered membrane lipid composition and packing could contribute to the aging phenotype, a hypothesis that we subsequently tested using plasma membrane-specific analyses.

Then, to test whether ELOVL2 dysfunction is sufficient to recapitulate aging-associated transcriptional signatures, we analyzed transcriptomic profiles from 18-month-old *Elovl2*^*C234W*^ mutant mice, which harbor a catalytically selective mutation that abolishes the conversion of 22:5n-3 to 24:5n-3 [11]. Gene set enrichment analysis revealed significant enrichment of plasma membrane raft, endocytic vesicle, membrane raft organization, and basement membrane categories relative to age-matched controls (Fig. 1E). In addition, pathways related to epithelial-to-mesenchymal transition (EMT) and complement activation were significantly enriched (Fig. 1E, F), indicating that ELOVL2 deficiency recapitulates both structural remodeling and inflammatory features associated with aging and RPE aging [19–21].

### Morphometric and ultrastructural remodeling of RPE in ELOVL2-deficient mice

**Fig. 2:**
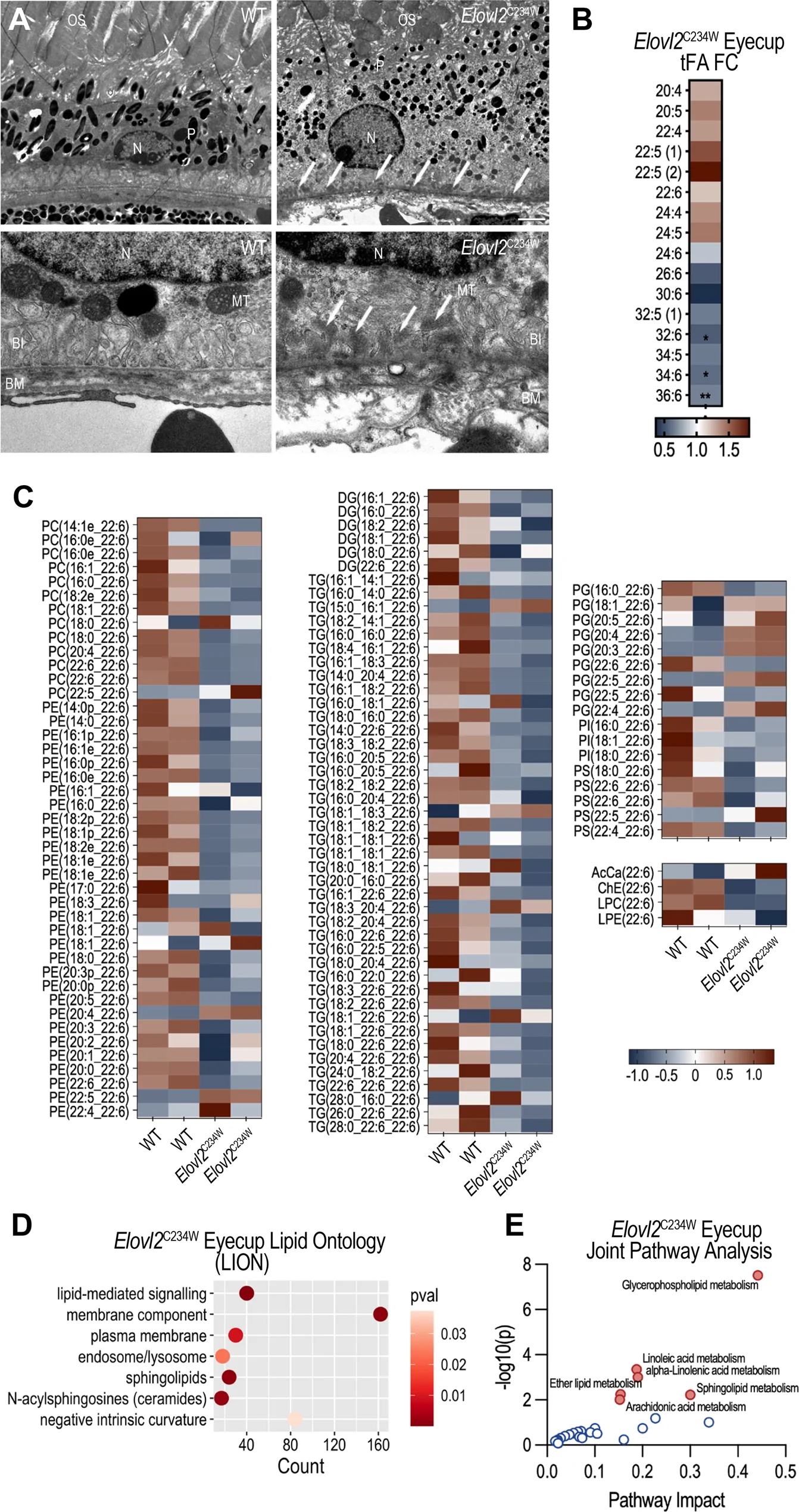
Disruption of LC-PUFA synthesis drives ultrastructural defects and lipid remodeling. (**A**) Transmission electron microscopy images of the RPE–photoreceptor interface from 18-month-old wild-type and *Elovl2*^C234W^ mice. OS, outer segments; P, pigment granules; N, nucleus; MT, mitochondria; BI, basal infoldings. White arrows indicate accumulation of amorphous sub-RPE material between the RPE and Bruch’s membrane. Scale bars, 2 μm (top) and 1 μm (bottom). (**B**) Heatmap showing fold changes in total fatty acid changes in eyecups from *Elovl2*^C234W^ mice relative to age-matched wild-type controls. Values represent the mean fold change of *n* = 3 animals per group. (**C**) Heatmap of DHA-containing lipid species in *Elovl2*^C234W^ eyecups (*n* = 2 per group). (**D**) Lipid Ontology (LION) enrichment analysis of significantly altered lipid species (*n* = 2 per group). (**E**) Joint pathway analysis integrating lipidomic and transcriptomic datasets using MetaboAnalyst. Statistical significance was determined using unpaired two-tailed t tests (**B**). Data are mean ± SEM. *p < 0.05, **p < 0.01, ***p < 0.001, ****p < 0.0001. Lipidomics data from mouse eyecups have been deposited in Dryad (DOI https://doi.org/10.5061/dryad.34tmpg51p) (**B-E**)

To determine whether these transcriptional and metabolic changes correspond to structural alterations, we performed morphometric analysis of RPE flatmounts from aged wild-type and *Elovl2*^*C234W*^ mice. Mutant RPE exhibited pronounced disruption of epithelial architecture and the presence of stress fibers (Fig. 1G), including loss of regular hexagonal organization and emergence of enlarged, circular, and poorly interdigitated cells (Fig. 1H). To quantify regional effects, RPE monolayers were subdivided into central, mid-peripheral, and peripheral zones and analyzed using the REShAPE pipeline (Fig. 1I). *Elovl2*^*C234W*^ mice showed region-dependent remodeling, including reduced central cell area, increased peripheral cell area, and increased aspect ratio across regions (Fig. 1I, S1B), consistent with aging-associated RPE stress phenotypes [22]. To further assess retinal consequences, two-photon excitation imaging with fluorescence lifetime imaging microscopy (FLIM) revealed subretinal accumulation of fluorescent puncta in *Elovl2*^*C234W*^ mice but not controls (Fig. S1C–D), consistent with accumulation of retinal condensation products. Transmission electron microscopy further revealed ultrastructural defects, including loss of basal infoldings and accumulation of amorphous sub-RPE material between the RPE and Bruch’s membrane (Fig. 2A), indicating impaired structural integrity of the RPE–choroid interface as observed during aging and in AMD [23, 24].

### Age- and ELOVL2-associated alterations of the RPE lipidome

Given the structural and transcriptional remodeling observed, we next assessed the extent to which aging and ELOVL2 deficiency alter lipid composition. Total fatty acid profiling revealed a consistent depletion of LC-PUFA species in both aged wild-type and *Elovl2*^*C234W*^ eyecups, indicating convergence on a shared PUFA-deficient state (Fig. 2B, S2A). Untargeted lipidomic profiling showed broad remodeling of lipid classes, including increased ceramides (Cer), sphingomyelins (SM), phosphatidylcholines (PC) and phosphatidylethanolamines (PE) (Fig. S2B, C). Consistent with impaired ELOVL2-mediated PUFA elongation, the abundance of both PUFA-containing and DHA-containing lipid species was reduced in 18-month-old *Elovl2*^C234W^ eyecups relative to wild-type animals, with DHA-containing lipids showing a particularly pronounced decrease (Fig. 2C, S2D). Lipid ontology analysis further revealed enrichment of lipid species associated with membrane structure, endosomal/lysosomal compartments, and lipid signaling (Fig. 2D. Joint pathway analysis (MetaboAnalyst [25]) integrating 18-month-old *Elovl2*^C234W^ eyecup transcriptomic and lipidomic datasets confirmed coordinated dysregulation of glycerophospholipid, sphingolipid, and PUFA metabolism pathways (Fig. 2E). Finally, direct comparison of 18-month-old wild-type and 15-month-old *Elovl2*^*C234W*^ samples revealed highly similar lipidomic profiles, including the abundance of PUFA containing and DHA containing lipid species (Fig. S2E, F). Pearson’s correlation analysis of all detected lipid species also showed near-perfect correlation between samples (*r* ≈ 1), indicating highly similar lipidomic profiles (Fig. S2G) congruent with accelerated aging phenotype of mutant RPE.

### ELOVL2 regulates plasma membrane lipid composition and biophysical properties in RPE cells

**Fig. 3:**
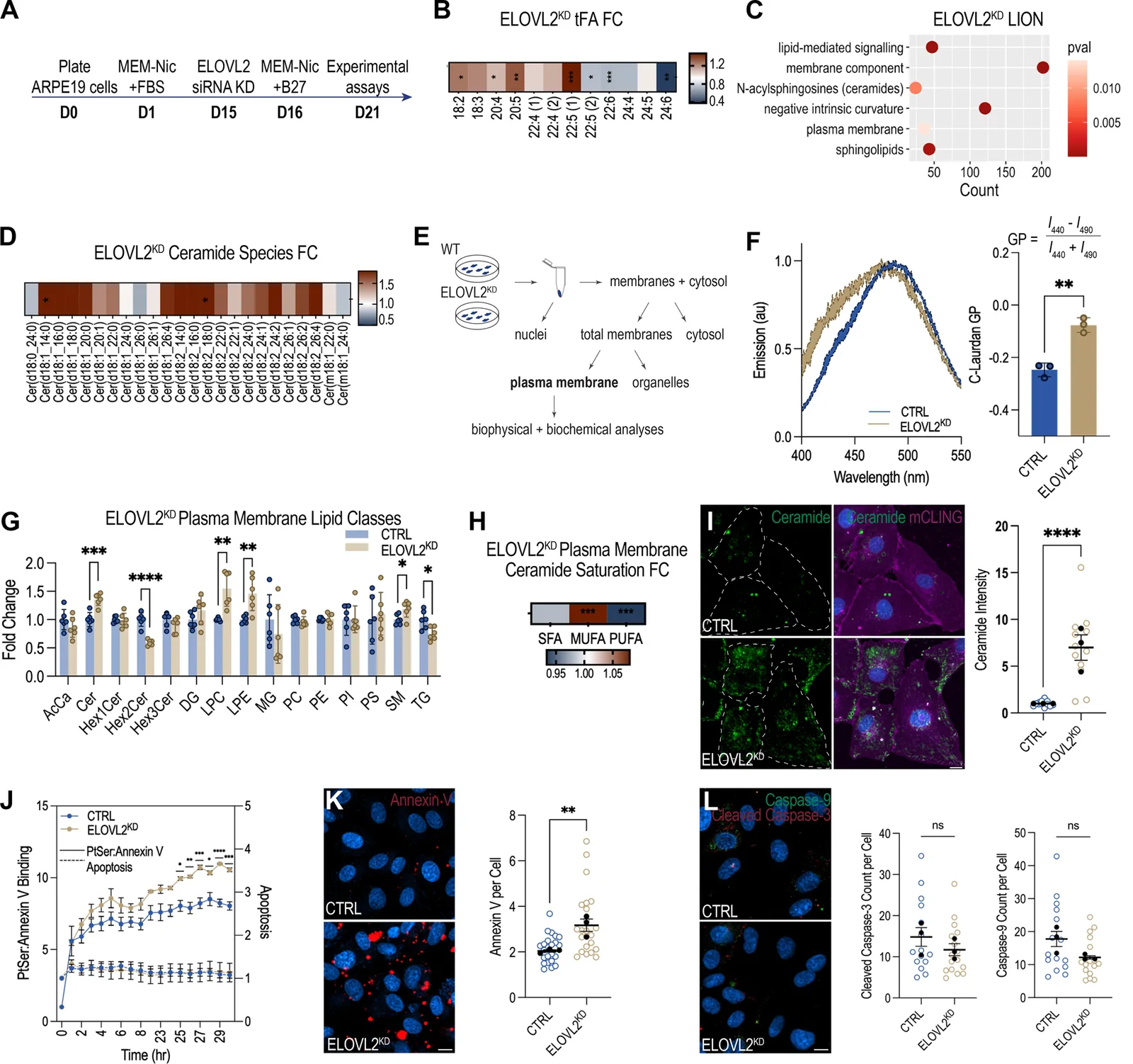
ELOVL2 loss remodels the plasma membrane lipidome and induces membrane stress. (**A**) Schematic of ARPE-19 differentiation and ELOVL2 knockdown (*ELOVL2*^KD^). (**B**) Heatmap of significantly altered total fatty acids in whole-cell lipidomics from *ELOVL2*^KD^ cells (*n* = 4 per group). (**C**) LION enrichment analysis of altered lipid species. (**D**) Heatmap of ceramide species (*n* = 4 per group). (**E**) Workflow for plasma membrane isolation. (**F**) C-Laurdan generalized polarization (GP) analysis. Left: representative spectra. Right: GP quantification (*n* = 3 per group). (**G**) Altered lipid classes in plasma membrane fractions (*n* = 6 per group). (**H**) Ceramide saturation profiles (SFA, MUFA, PUFA) in plasma membrane fractions (*n* = 6 per group). (**I**) Left: representative images of mCLING (pink) and ceramide (green). Right: quantification. Data are normalized replicate mean ± SEM (closed circles; *n* = 3) with individual fields (open circles; *n* = 4 per replicate). Scale bars, 10 μm. (**J**) Real-time phosphatidylserine externalization (Annexin V, solid lines) and apoptosis activation (dashed lines) (*n* = 3 per group). (**K**) Annexin V staining and quantification. Data are normalized replicate mean ± SEM (closed circles; *n* = 3) with individual fields (open circles; *n* > 6 per replicate). (**L**) Caspase-9 and cleaved caspase-3 staining and quantification. Data are normalized replicate mean ± SEM (closed circles; *n* = 3) with individual fields (open circles; *n* = 4 per replicate). Statistical significance was determined using unpaired two-tailed t tests (B, D, F to I, K, L) or two-way ANOVA (**J**). Data are mean ± SEM. *p < 0.05, **p < 0.01, ***p < 0.001, ****p < 0.0001. Lipidomics data from *ELOVL2*^KD^ cells have been deposited in Dryad (DOI https://doi.org/10.5061/dryad.34tmpg51p) (**B-D, G, H**)

To directly test the role of ELOVL2 in regulating lipid composition of RPE membranes, we performed siRNA-mediated knockdown in differentiated ARPE-19 cells. ARPE-19 cells were differentiated in 10 nM nicotinamide [26], followed by siRNA-mediated knockdown of *ELOVL2* and 6 d in FBS-depleted media supplemented with B27 to provide essential fatty acids, vitamins, antioxidants, and growth factors while minimizing confounding exogenous PUFA sources (Fig. 3A, S3A). ELOVL2 knockdown led to accumulation of substrate fatty acids and depletion of downstream long-chain PUFA products, confirming impaired elongase activity (Fig. 3B, S3B). Lipidomic analysis revealed coordinated remodeling of membrane-associated lipid classes, including increased sphingomyelins and phospholipids and a redistribution of neutral lipids (Fig. S3C, D). Lipid ontology analysis confirmed enrichment of membrane and signaling-associated lipid species consistent with in vivo findings (Figs. 3C, 2D). Notably, ceramide species were significantly increased (Fig. 3D, S3E), indicating a shift toward a membrane composition associated with increased rigidity and stress susceptibility.

Our structural and lipidomic data suggested significant PUFA-dependent alterations in RPE plasma membrane (Fig. 1G–I). To directly assess the effects of lipid remodeling on plasma membrane properties, we next isolated plasma membrane fractions and, after confirming their purity (Fig. 3E, S3F), characterized their biophysical properties and lipid composition. C-Laurdan, a membrane-phase-sensitive fluorescent dye [27], analysis demonstrated significantly increased generalized polarization (GP) in ELOVL2-deficient cells, indicating increased membrane order and decreased fluidity (Fig. 3F). Plasma membrane lipidomics further confirmed enrichment of long-chain ceramides, characteristic for the retina, lysophospholipids and a shift toward MUFA-ceramides with a corresponding decrease in PUFA-ceramides, a pattern similar to the ceramide saturation profile observed in aging eyecups (Fig. 3G, H, S3G). Consistently, ceramide accumulation at the cell surface, stained with membrane-binding fluorophore-cysteine-lysine-palmitoyl group (mCLING), was confirmed by immunofluorescence (Fig. 3I).

### ELOVL2-deficient RPE cells exhibit plasma membrane stress without apoptosis

Given the altered plasma membrane biophysical properties and composition, we next assessed membrane stress in ELOVL2-deficient cells by measuring the loss of lipid bilayer asymmetry. We measured externalized phosphatidylserine (PS), an early marker of membrane stress and apoptosis, using two complementary Annexin V binding assays. ELOVL2 deficiency resulted in increased PS externalization, indicating plasma membrane perturbation (Fig. 3J, K).

However, this was not accompanied by activation of apoptotic markers, including cleaved caspase-3 or caspase-9 (Fig. 3L), in contrast to cells treated with H_2_O_2_ (6 h) (Fig. S3J, K). These results indicate that cells maintain viability despite persistent membrane stress.

### Lysosome-mediated plasma membrane repair is activated in response to membrane stress

**Fig. 4:**
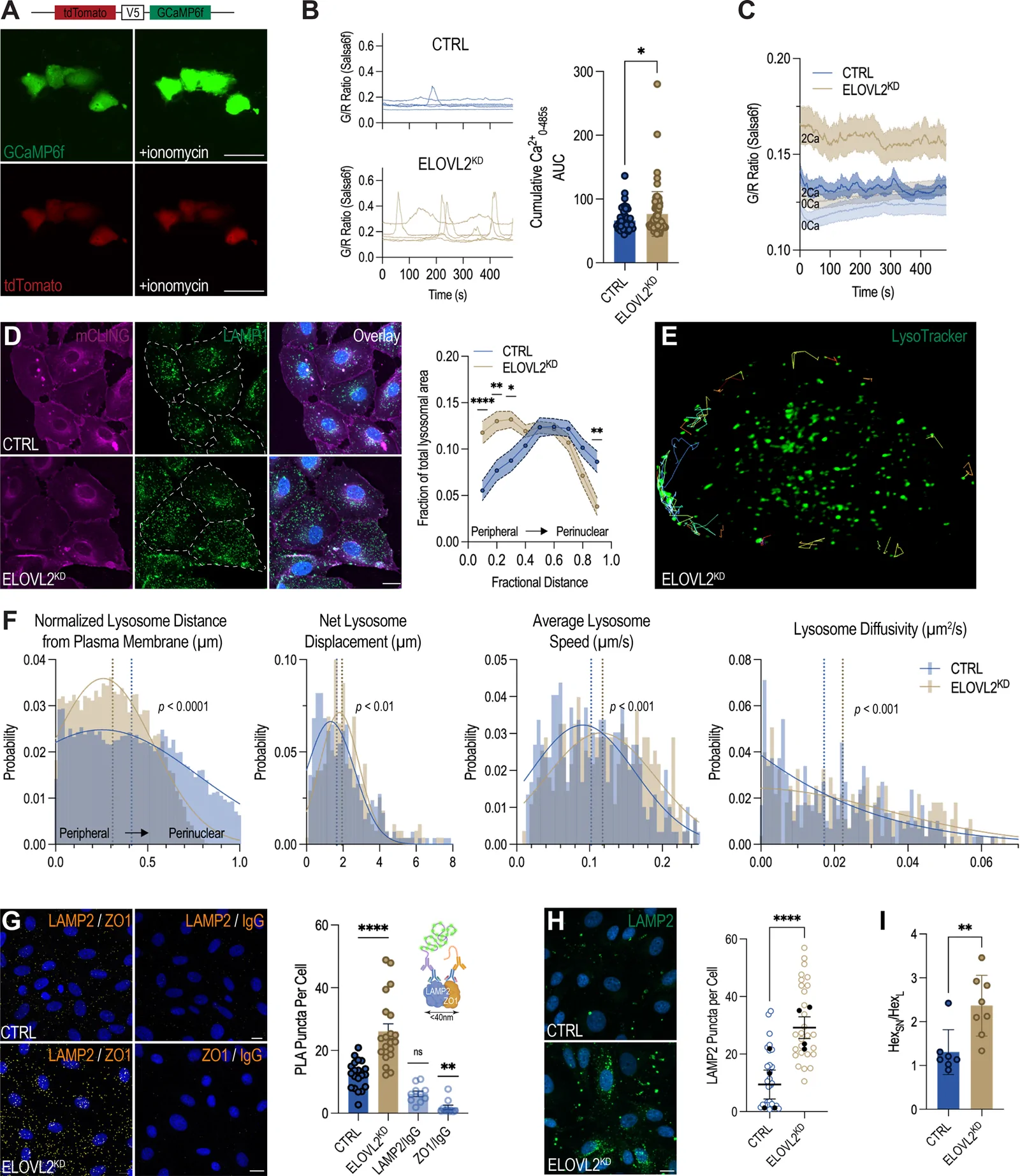
ELOVL2 deficiency alters lysosomal dynamics and lysosome–plasma membrane fusion. (**A**) Validation of Salsa6f calcium indicator. Representative images before and after ionomycin stimulation. Scale bars, 50 μm. (**B**) Calcium traces from control and *ELOVL2*^KD^ cells and quantification by area under the curve. (**C**) Averaged calcium traces under 0 Ca^2+^ and 2 Ca^2+^ conditions. (**D**) Lysosome distribution analysis. Left: representative images. Right: radial distribution quantification. Scale bars, 10 μm. (**E**) Lattice light-sheet microscopy of lysosome tracking near the plasma membrane. (**F**) Histogram distribution of lysosome dynamics, including distance, displacement, speed, and diffusivity. Distributions were fitted to Gaussian functions, with dashed lines indicating medians. (**G**) Lysosome–plasma membrane proximity ligation assay (PLA). Left: representative images. Right: quantification. Data are mean ± SEM (*n* = 20 fields in each group, with >8 cells per field, pooled from two biologically independent experiments). Scale bars, 10 μm. (**H**) Surface LAMP2 detection in nonpermeabilized cells and quantification. Data are replicate mean ± SEM (closed circles; *n* = 4) with individual fields (open circles; *n* > 6 per replicate). (**I**) Lysosomal exocytosis measured by β-hexosaminidase release. Statistical significance was determined using unpaired two-tailed t tests (**B, H, I**), two-way ANOVA (**D**), Mann–Whitney U test (**F**), or one-way ANOVA with Tukey’s test (**G**). Data are mean ± SEM. *p < 0.05, **p < 0.01, ***p < 0.001, ****p < 0.0001

We next asked how ELOVL2-deficient cells maintain viability under conditions of membrane stress. Live-cell calcium imaging revealed elevated cytosolic Ca^2+^ levels in ELOVL2-deficient cells in standard extracellular 2 mM [Ca^2+^] conditions, consistent with increased plasma membrane permeability (Fig. 4A, B). Comparing average calcium levels between the 2Ca and 0Ca conditions readily revealed significantly elevated baseline levels in *ELOVL2*^KD^ cells compared to control cells (Fig. 4C, S4A). Disruption of the calcium gradient across the plasma membrane may promote the mobilization of lysosomes toward the plasma membrane to support membrane repair [28–30]. LAMP1 immunostaining revealed redistribution of lysosomes toward the cell periphery in ELOVL2-deficient cells (Fig. 4D, S4B) while no significant changes were observed in lysosomal number or diameter (Fig. S4C, D). To visualize lysosome dynamics, cells were imaged using 4D adaptive optics lattice light-sheet microscopy (Fig. 4E). Lysosome positions were tracked and analyzed relative to cell boundaries, and dynamic parameters were calculated from time-lapse image sequences using particle-tracking software [31]. These results further confirmed increased lysosome accumulation and motility near the plasma membrane (Fig. 4F), indicating enhanced lysosomal engagement with the cell cortex. Proximity ligation assays, using LAMP2 and ZO-1 antibodies, demonstrated increased lysosome–plasma membrane contact (Fig. 4G), and surface exposure of LAMP2 confirmed lysosomal fusion at the plasma membrane (Fig. 4H). Consistently, β-hexosaminidase release into the extracellular medium was significantly increased in ELOVL2-deficient cells, confirming lysosomal exocytosis (Fig. 4I). These data suggest that ELOVL2 depletion leads to lysosomal exocytosis in response to plasma membrane stress. Because lysosomal trafficking is redistributed to membrane repair, we next examined whether this affects RPE function. In vivo, wild-type animals exhibited robust accumulation of internalized 1D4-positive phagosomes at the earliest time point (ZT1), followed by a progressive decline at later time points (ZT4/ZT8) as phagosomes were cleared (Fig. S4G). In contrast, *Elovl2*^*C234W*^ mutants displayed significantly fewer internalized phagosomes (ZT1), indicating reduced POS ingestion during the peak of the phagocytic burst (Fig. S4G). Furthermore, phagosome size was significantly larger in mutants, consistent with delayed processing or stalled lysosomal degradation (Fig. S4G). In vitro, ELOVL2 knockdown similarly reduced uptake of fluorescently labelled outer segments (Fig. S4H), indicating impaired phagocytic capacity in both systems.

### ELOVL2 deficiency triggers membrane stress and polarized lysosomal responses in iPSC-RPE

**Fig. 5:**
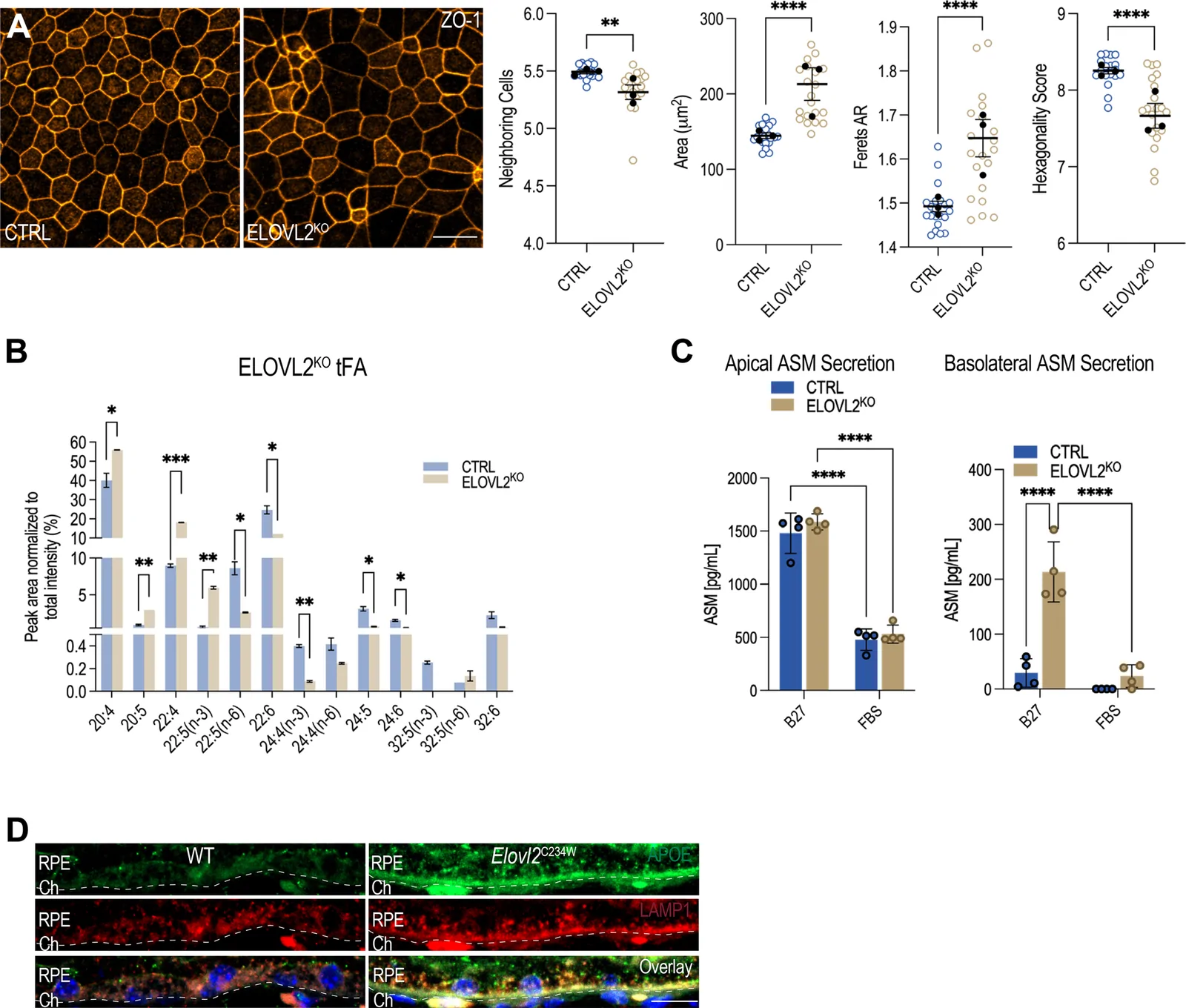
ELOVL2 depletion induces basolateral lysosome–plasma membrane remodeling. (**A**) ZO-1–labeled tight junctions in control and *ELOVL2*^KO^ iPSC-RPE monolayers and morphometric analysis. Data are normalized replicate mean ± SEM (closed circles; *n* = 3) with individual fields (open circles; *n* > 6 per replicate). Scale bars, 20 μm. (**B**) Relative abundances of LC- and VLC-PUFAs (*n* = 2 per group). (**C**) Acid sphingomyelinase levels in apical and basolateral compartments (*n* = 4 per group). (**D**) Retinal sections stained for APOE and LAMP1. Scale bars, 10 μm. Statistical significance was determined using unpaired two-tailed t tests. Data are mean ± SEM. *p < 0.05, **p < 0.01, ***p < 0.001, ****p < 0.0001. Lipidomics data from *ELOVL2*^KO^ iPSC-RPE have been deposited in Dryad (DOI https://doi.org/10.5061/dryad.34tmpg51p) (**B**)

To validate these findings in a human model, we generated ELOVL2 knockout iPSC-derived RPE. Differentiation status was assessed by qPCR, confirming robust expression of canonical RPE markers—including *PAX6*, *RPE65*, *GAS6*, and *MFGE8*—while *VEGFA* and *MERTK* showed modest reductions in *ELOVL2*^KO^ cells, along with a marked decrease in *ELOVL2* expression (Fig. S5A). Similar to previous findings in vivo, morphometric analysis of *ELOVL2*^KO^ cells revealed disrupted epithelial morphology (Fig. 5A). Fatty acid profiling confirmed accumulation of ELOVL2 substrates and depletion of downstream PUFA products (Fig. 5B), consistent with impaired elongation activity.

Next, we examined whether the process of lysosomal exocytosis contributes to plasma membrane remodeling in polarized iPSC-RPE cells by measuring acid sphingomyelinase (ASM) release by basal and apical membranes. First, we found that ASM release was higher in the apical than the basolateral compartment under lipid-rich (FBS) and essential fatty acid only (B27) media conditions. Second, overall ASM secretion was greater in both CTRL and *ELOVL2*^KO^ cells in DHA-depleted (B27) medium compared to FBS medium. Third, *ELOVL2*^KO^ cells exhibited a selective increase in basolateral ASM release in B27 medium, which was significantly decreased when cells were cultured in a PUFA-rich (FBS) medium (Fig. 5C), indicating directional lysosomal exocytosis in response to membrane stress. Using a lipidomics-based phagocytosis assay [32], we found that *ELOVL2*^KO^ cells also exhibited decreased internalization of POS (Fig. S5B). Consistent with broader defects in degradative pathways, *ELOVL2*^KO^ iPSC-RPE also exhibited a decreased LC3-II to LC3-I ratio, indicative of reduced autophagosome formation and impaired autophagy [33] (Fig. S5C). Finally, in vivo analysis revealed basal redistribution of LAMP1 and increased APOE accumulation at the RPE–Bruch’s membrane interface in *Elovl2*^*C234W*^ mice (Fig. 5D), suggesting coordinated basal membrane remodeling and extracellular deposition.

### ELOVL2-dependent lipid products directly regulate membrane stress and lysosome activity

**Fig. 6:**
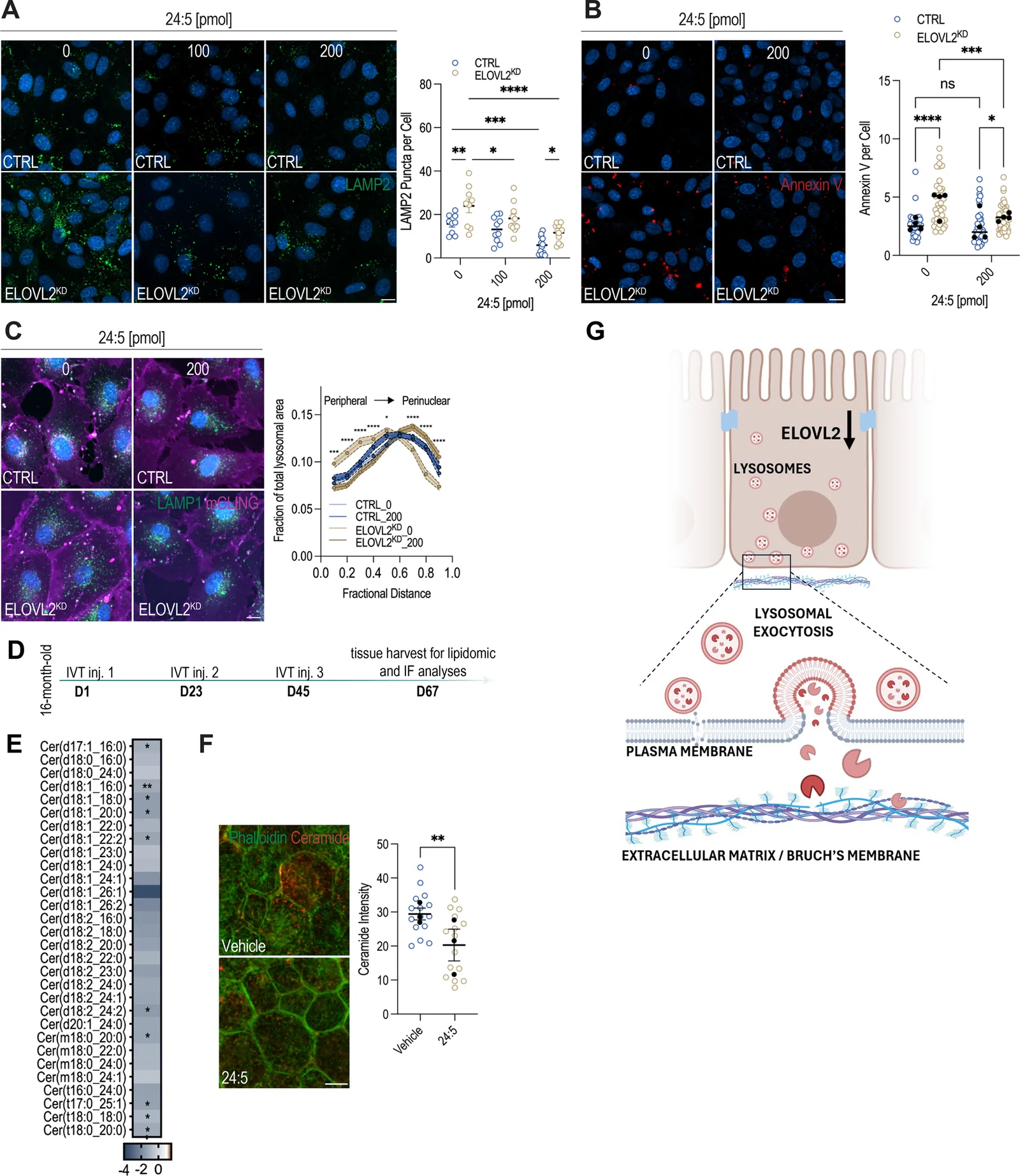
Supplementation with an ELOVL2 product rescues age-associated phenotypes. (**A**) Surface LAMP2 staining in control and *ELOVL2*^KD^ cells treated with 24:5n-3 and quantification. Data are replicate mean ± SEM (closed circles; *n* = 3) with individual fields (*n* > 8 fields per condition), pooled from two biologically independent experiments. Scale bars, 10 μm. (**B**) Annexin V staining and quantification following 24:5n-3 treatment. Data are normalized replicate mean ± SEM (closed circles; *n* = 4) with individual fields (open circles; *n* > 9 per replicate). (**C**) Lysosome distribution following 24:5n-3 treatment. (**D**) Experimental design for intravitreal supplementation. (**E**) Heatmap of ceramide species in supplemented mice (*n* = 3 per group). (**F**) RPE flat mounts stained for ZO-1 and ceramide and quantification. Data are replicate mean ± SEM (closed circles; *n* = 3) with individual fields (open circles; *n* = 5 per replicate). Scale bars, 10 μm. (**G**) Model of membrane remodeling in aging RPE. Statistical significance was determined using two-way ANOVA (**A**, **B**, **C**) or unpaired two-tailed t tests (**E, F**). Data are mean ± SEM. *p < 0.05, **p < 0.01, ***p < 0.001, ****p < 0.0001. Lipidomics data from 24:5n-3-supplemented mouse eyecups have been deposited in Dryad (DOI https://doi.org/10.5061/dryad.34tmpg51p) (**E**)

To test whether restoration of ELOVL2 enzymatic products can reverse membrane stress, we supplemented cells for 4 days with the direct product of ELOVL2, 24:5n-3, and revealed reduced surface exposure of LAMP2 and PS externalization in both control and ELOVL2-deficient cells (Fig. 6A, B), indicating direct dependence of membrane stress on ELOVL2 activity. Consistent with reduced membrane stress, 24:5n-3 supplementation also altered lysosomal positioning, shifting lysosome distribution in ELOVL2^KD^ cells from a predominantly peripheral localization to a more perinuclear pattern (Fig. 6C). In vivo, intravitreal 24:5n-3 supplementation reduced ceramide accumulation and restored lipid balance in aged eyecups (Fig. 6D, E, F), demonstrating that lipid remodeling is reversible and directly linked to ELOVL2 metabolic output.

## Discussion

Here, we identify a central role for ELOVL2-mediated PUFA elongation in maintaining plasma membrane composition, biophysical properties, and structural integrity in the RPE, a specialized neuroepithelial tissue. Loss of ELOVL2 activity disrupts lipid balance, driving ceramide accumulation and depletion of long-chain PUFA-containing lipids, shifting plasma membrane properties toward a more ordered state. This membrane stress is associated with Ca^2+^-dependent recruitment of lysosomes to the cell periphery, where they engage in a compensatory repair response (Fig. 6G). We propose a model in which age-related lysosome-mediated membrane repair is a potential mechanism by which age-related chronic lipid imbalance is accommodated in the RPE.

Our analysis of human RPE across the lifespan revealed a consistent enrichment of plasma membrane and endomembrane-related genes, indicating that membrane homeostasis is altered during aging. Complementary analyses of aged mouse eyecups demonstrated similar membrane remodeling signatures. These findings, together with lipidomic profiling, support a concept in which age-associated metabolic changes converge on membrane composition as a potential primary site of vulnerability.

PUFAs are critical determinants of membrane properties, and altered PUFA homeostasis has been linked to AMD risk and aging retina [34–36]. Consistently, loss of PUFA elongation in eyecups from *Elovl2*^*C234W*^ mutant mice resulted in a marked accumulation of ceramides and sphingomyelins alongside depletion of PUFA species (Fig. 2B, S2A–D). These compositional changes are consistent with increased lipid packing and reduced membrane fluidity (Fig. 3F–H). Accordingly, depletion of PUFA-containing phospholipids impairs membrane-dependent processes, including phagocytosis, autophagy, and barrier maintenance. Similar disruptions in PUFA homeostasis have been reported in AdipoR1 deficiency models, where ceramide accumulation and reduced omega-3 PUFA levels are associated with retina and RPE dysfunction. Importantly, experimental manipulation of ceramide levels in these models partially rescued these phenotypes [37–39]. Together, these findings suggest a conserved relationship between PUFA metabolism, ceramide balance, and membrane biophysical integrity in aging tissues of the central nervous system.

Remarkably, despite clear evidence of membrane stress, including PS externalization, ELOVL2-deficient RPE cells did not progress to apoptosis. Instead, they activated a Ca^2+^-dependent lysosomal response detected by lysosome redistribution, elevated surface exposure of LAMP2, and increased release of lysosomal enzymes including β-hexosaminidase and ASM (Fig. 4B–I, S4F, Fig. 5C). These observations suggest activation of a lysosome-dependent plasma membrane repair pathway that preserves cell viability under conditions of chronic lipid imbalance. While lysosomal exocytosis has been described in RPE responses to acute stressors such as sublytic complement attack [40, 41], our findings suggest that age-associated lipid remodeling can engage this repair pathway independently of acute injury. Notably, the observed response here is preferentially oriented toward the basolateral surface, suggesting spatial regulation of membrane repair in polarized epithelia.

This mechanism is conceptually distinct from classical lysosomal storage disorders, in which defects lead to secondary lipid accumulation and membrane dysfunction [42]. In contrast, our data supports an inverse model in which lipid imbalance initiates compensatory lysosomal engagement. Similarly, peroxisomal disorders share conceptual overlap where impaired DHA synthesis due to defective peroxisomal β-oxidation perturbs membrane composition [43]. The mechanistic origin of lipid imbalance, however, differs fundamentally. In ELOVL2 deficiency, organelle function seems to be largely unaffected.

Although lysosome-mediated repair preserves membrane integrity, chronic activation of this pathway has downstream consequences for tissue remodeling. Specifically, increased secretion of ASM during lysosomal exocytosis provides a mechanistic link between intracellular membrane stress and extracellular lipid remodeling. ASM hydrolyzes sphingomyelin to ceramide at the cell surface, generating membrane domains with negative curvature, as previously observed in diseased RPE [37–39, 44–46], that can further promote membrane rupture [47, 48]. Consistently, we observed increased levels of both sphingomyelin and ceramide in plasma membrane fractions, supporting enhanced substrate availability and local ceramide production (Fig. 3G). Elevated basolateral ASM secretion in ELOVL2-deficient RPE thus represents a coordinated, but potentially maladaptive, response to the membrane stress.

The polarized nature of lysosomal exocytosis further suggests that membrane stress responses are spatially organized and may differentially impact tissue compartments. Basolateral release of lysosomal contents, including undigested material and enzymes involved in protein and lipid degradation, provides a possible mechanism for the age-related Bruch’s membrane remodeling and accumulation of sub-RPE deposits. Consistent with this model, we observed increased APOE accumulation at the basal RPE surface in *Elovl2*^*C234W*^ mice (Fig. 5D), linking intracellular membrane stress to extracellular lipid deposition and features reminiscent of early AMD and age-associated neurodegeneration.

Our findings also suggest that environmental factors, such as nutrient availability, can modulate this pathway. We show that lipid restriction induces membrane stress and lysosomal exocytosis in wild-type RPE cells, suggesting that reduced access to exogenous lipids, such as may occur in the presence of sub-RPE deposits, can exacerbate membrane instability. This raises the possibility that metabolic and environmental stressors converge on a shared membrane maintenance pathway during aging.

More broadly, these findings position lysosomes as central regulators of membrane homeostasis in aging cells. Beyond their canonical degradative roles, lysosomes mediate membrane repair, nutrient sensing, and stress adaptation [49–54]. While lysosomal degradative capacity declines in aging, lysosomal exocytosis may increase as a compensatory response to membrane stress.

Although the role of ELOVL2 in the RPE has not been extensively characterized, the RPE depends on ELOVL2-mediated PUFA synthesis to maintain membrane composition and support photoreceptor function [32, 39, 55–58]. Consistent with this, supplementation with 24:5n-3 restored membrane properties, reduced ceramide accumulation, and attenuated lysosome-mediated remodeling, directly linking ELOVL2 enzymatic activity to membrane homeostasis.

Together, our findings identify age-associated lipid remodeling driven by reduced ELOVL2 activity as a main mechanism of plasma membrane dysfunction in the RPE. Loss of ELOVL2-dependent PUFA elongation depletes long-chain PUFA-containing phospholipids, promotes ceramide accumulation, resulting in increased plasma membrane order and structural instability. This triggers a Ca^2+^-dependent lysosome repair program that preserves cell viability but may contribute to chronic extracellular remodeling at the RPE–Bruch’s membrane interface, features reminiscent of early AMD. Restoration of ELOVL2-derived product 24:5n-3 reverses these defects, linking membrane homeostasis directly to ELOVL2-dependent lipid metabolism. Collectively, our findings establish ELOVL2-dependent lipid metabolism as a key regulator of plasma membrane integrity and identify lysosome-mediated membrane repair as a fundamental adaptive mechanism linking metabolic aging to epithelial dysfunction.

## Methods

### Cell culture

#### ARPE-19

ARPE-19 (ATCC CRL-2302) cells were maintained in DMEM/F12 + GlutaMAX supplemented with 10% FBS at 37 °C in a humidified incubator with 5% CO_2_. For differentiation, ARPE-19 cells were cultured in MEM-Nic medium (MEM alpha with GlutaMAX, 1% FBS, 1% Penicillin/Streptomycin, 1% N1 supplement, taurine (0.25 mg/mL), hydrocortisone (20 ng/mL), triiodothyronine (0.013 ng/mL), and 10 mM nicotinamide) with media changes three times per week for two weeks following a previously published protocol [26].

To reduce the source of exogenous lipids, cells were cultured in a defined minimal medium consisting of the same MEM-Nic media used for standard maintenance with chemically defined, serum-free supplement B27 (Gibco). Cells were maintained in B27 medium for 6 d prior to downstream assays.

#### iPSC-RPE

##### Cell culture media and composition

N2 supplement (1x). 100 µg/mL Optiferrin (Invitria, #777TRF029), 5 µg/mL insulin (Sigma, #4512), 6.3 ng/mL progesterone (Sigma, #P8783), 16.11 µg/mL putrescine (Sigma, #P5780), 5.2 ng/mL sodium selenite (Sigma, #S9133) in DMEM/F12 (Gibco #11330-057) as previously described [59].

B8 stem cell medium. DMEM/F12 with HEPES (Corning #10-092-CV), 200 µg/ml L-Ascorbic acid 2-phosphate trisodium salt (Fujifilm Wako, #011-28471), 5 µg/ml insulin (Sigma #4512), 5 µg/ml Optiferrin (Invitria #777TRF029), 20 ng/ml sodium selenite (Sigma #S9133), 40 ng/ml fibroblast growth factor 2-G3 (FGF2-G3) (Qkine #Qk053), 0.1 ng/ml neuregulin 1 (NRG1; Peprotech #100–03), 0.1 ng/ml transforming growth factor beta-1 (TGFβ1; Qkine, #Qk010) as previously described [60].

B27 Supplement for IPSCs (1x). B27 medium was prepared according to its original formulation, with minor modifications [61]. 2.5 mg/mL bovine serum albumin - BSA (FA and IgG-free, Fraction V; Sigma #A4919), 2.5 µg/mL catalase (Sigma #C40), 2.5 µg/mL superoxide dismutase (Sigma #S5395), 3.125 µg/mL human recombinant insulin (Sigma #4512), 5 µg/mL Optiferrin (Invitria #777TRF029), 1.0 µg/mL reduced glutathione (Sigma# G6013), 16.1 µg/mL putrescine-HCl (Sigma #P5780), 14.35 ng/mL sodium selenite (Sigma# S5261), 2 ng/mL triiodothyronine (Sigma #T6397), 6.3 ng/mL progesterone (Sigma #P8783), 20 ng/mL corticosterone (Sigma #C2505), 2.0 µg/mL L-carnitine (Sigma #C0283), 1.0 µg/mL ethanolamine (Sigma #E9508), 15 µg/mL D-galactose (Sigma #G0625), 1.0 µg/mL linoleic Acid (Sigma #L1012), 1.0 µg/mL linolenic Acid (Sigma #L2376), 47 ng/mL lipoic acid (Sigma #T1395), 100 ng/mL retinyl acetate (Sigma #R7882), 1 µg/mL ɑ-tocopherol (Sigma #T3251), 1.0 µg/mL ɑ-tocopherol Acetate (Sigma #T3001), 10 ng/mL biotin (Sigma #B4639), 1.0 µg/mL pipecolic acid (Sigma #P2519).

RIM (Retinal Induction Medium). DMEM/F12 (Gibco #11330), 200 µM ascorbic acid (Sigma#A4544), 1× B27 Supplement, 1.5% Knockout Serum Replacement (KSR; Gibco #10828010), 1x non-essential amino acids (NEAA; Gibco #11140050), 1× N2 supplement, 1x sodium pyruvate (Gibco #11360-070), 10 ng/mL AF-IGF-1 (Qkine #Qk047), 5 µM CKI-7 dihydrochloride (Sigma #1177141-67-1), 100 nM LDN-193189 (Med Chem Express #HY-12071A), 10 µM SB431542 (Ambeed #A172016).

RDM (Retinal Differentiation Medium). DMEM/F12 (Gibco #11330-057), 200 µM ascorbic acid, 1× B27 Supplement, 1.5% KSR, 1x NEAA, N2 supplement, 1x sodium pyruvate, 10 ng/mL AF-IGF-1 (Qkine #Qk047), 5 µM CKI-7 dihydrochloride (Sigma#1177141-67-1), 100 nM LDN-193189 (Med Chem Express #HY-12071A), 1 µM PD0325901 (Med Chem Express #HY-10254), 10 µM SB431542 (Ambeed #A172016).

RMNA (Retinal Media with Nicotinamide and Activin-A). DMEM/F12 (Gibco #11330-057), 200 µM ascorbic acid (Sigma #A4544), 1× B27 Supplement, 1.5% KSR, 1x NEAA, 1× N2 supplement, 1x sodium pyruvate, 100 ng/mL Activin-A (Qkine #Qk001), 10 mM nicotinamide (Sigma #N0636).

RPE-MM (RPE maturation media). MEM alpha (Gibco #12571-063), 1x non-essential amino acids (NEAA; Gibco #11140050), 1× N2 supplement, 1x sodium pyruvate, 5% value grade heat-inactivated Fetal Bovine Serum (Gibco #A5256701), 13 ng/mL T3 (Sigma #T5516), 250 µg/mL taurine (Sigma #T8691).

##### Stem cell maintenance

EP1.1 iPSCs (female) [62] were used for the following study with approval from the UC San Diego and UC Irvine Institutional Review Boards. Stem cells were maintained antibiotic-free on 1% (vol/vol) Matrigel (MG)-GFR™ (Corning #354230) coated dishes at 37 °C under normoxia (5% CO_2_/20%O_2_) in mTSR1 or B8 media [60] as previously described. Cells were clump passaged every 4–6 d, with 0.5 mM EDTA in PBS for 7 min. After 24–48 h, cells were fed with mTSR1 or B8 alone.

*Creation of knockout iPSC lines*. ELOVL2 knockout was achieved EP1.1 iPSCs as previously described [63, 64]. Briefly, iPSCs were dissociated using Accutase (Sigma #A6964) for 12 minutes at 37 °C. After a brief centrifugation, the media was removed, and the cells were incubated on ice for 15 minutes. Immediately before electroporation, cells were resuspended in homemade R-buffer (Sucrose-based buffer: 250 mM sucrose and 1 mM MgCl_2_ in Ca^++^/Mg^++^ free DPBS (Gibco # 14,190)) containing RNPs targeting ELOVL2. Electroporation was performed at 1,300 V; interval, 20 ms; 1 pulse using the Neon Transfection system (Invitrogen). Cells were immediately transferred to 1% Matrigel-coated plates in B8 supplemented with 2 μM thiazovivin (Cayman Chemical #14245) to improve cell survival. For each genomic modification, RNPs for gene-editing were comprised of 4 µg of Cas9 protein (IDT) and ~0.4 µg of RNA targeting the *ELOVL2* gene (IDT). Electroporated iPSCs were single-cell passaged for clonal isolation, followed by genomic DNA extraction with Quick Extract (Biosearch Tech-formerly Lucigen), PCR amplification of the target region with Phusion Polymerase (Thermo # F548L), and oligos flanking the insert site (Forward: TTC CTA CTC AAA CCT TGA ACA GAT GCC AGA and Reverse: ACT TGG TAG AGG CAT CTG CTA CGT GGA TGT). Amplicons were Sanger sequenced with the nested primer (GCC CTC TAT CTG GAA GGA GAA) and aligned using Geneious Prime (Biomatters).

##### Generation of RPE from iPSC

To generate RPE from iPSC, cells were dissociated with TrypLE [65] for 10 min, quenched in B8T, and centrifuged for 5 min at 80 g as previously described [65]. The cell pellet was resuspended in 500 µL of B8T, counted by a hemocytometer, and plated into a Matrigel-coated 12-well TC-treated plate at a density of 110,000 cells per well in B8 plus 2 µM thiazovivin. The following day, B8T was aspirated and replaced with B8 media. Day 0 begins 48 h after the initial seeding of cells. From D0 to D2, cells were fed each day with Retinal Induction Media (RIM). From days 2 through 10, cells were fed Retinal Differentiation Media (RDM) daily. From day 11 to day 20, cells were fed with Retinal Media with Nicotinamide and Activin-A (RMNA). From D21 onwards, cells were fed with RPE Maturation Media (RPE-MM) until pigmented clusters emerged, typically around day 30. To enrich pigmented cell count, non-pigmented cells were scraped away with a flame-sterilized glass rod. Regions enriched for pigmented cells were carefully lifted off the plate using a flame-sterilized tungsten needle, gently aspirated using a P200 pipette, and transferred to a fresh TC-treated 1% Matrigel-coated 12-well plate in RPE-MM plus 2 µM thiazovivin. The following day, media was replaced with RPE-MM without thiazovivin and fed every other day from then on. These colonies were allowed to expand for 2–3 weeks, and then passaged with TrypLE into 12-well plates at a density of 100,000 cells per well. RPE was passaged each month (but no longer than two months as needed), up to passage 5, until enough RPE cells were present for plating on the required number of 12-well, 0.4 µm pore-size Transwell plates (Corning #3460) used for RPE maturation. For Transwells, cells were passaged with TrypLE as described above and plated at a density of 200,000 cells per well in the Matrigel-coated Transwell insert in RPE-MM plus thiazovivin. Media was replaced the following day with RPE media lacking thiazovivin and fed every other day for a total of 9 months from the first passage to the time of analysis in Transwell plates.

### Electroporation

ARPE-19 cells and differentiated ARPE-19 cells were transfected using the Neon Transfection System (Thermo Fisher Scientific) according to the manufacturer’s instructions. Briefly, cells were resuspended in Neon Resuspension Buffer R and electroporated with either a scrambled control siRNA or an *ELOVL2*-targeting siRNA (10 nM). Electroporation was performed using the Neon 10 µL or 100 µL tip with the following optimized parameters for ARPE-19 cells: 1350 V, 20 ms pulse width, 2 pulses. Immediately after electroporation, cells were transferred into pre-warmed culture medium and allowed to recover under standard culture conditions (37 °C, 5% CO_2_). The next day, the medium was replaced with serum-depleted medium containing B27 supplement.

### Bulk RNA sequencing

#### Sample collection and preparation

Fresh mouse eyecups were dissected from each mouse eye. Total RNA was extracted using the RNeasy Plus Mini Kit (Qiagen) following the manufacturer’s instructions. RNA quantity and quality were assessed using the Qubit RNA HS Assay kit (Thermo Fisher Scientific) and the Agilent Bioanalyzer RNA 6000 Pico kit (Agilent Technologies), respectively. RNA integrity was evaluated using RNA integrity number (RIN) values, and samples meeting the quality criteria (RIN ≥ 7.0) for library preparation were used for sequencing.

#### Library construction and sequencing

RNA sequencing libraries were prepared using the Illumina TruSeq RNA Library Prep kit. Paired-end sequencing was performed on the NovaSeq 6000 System using the Flow Cell Type S4, generating paired-end reads with a length of 100 base pairs and approximately 30 million reads per sample.

#### Bioinformatics analysis

Raw sequencing reads were preprocessed to remove adapter sequences and low-quality bases using Trimmomatic. Cleaned reads were mapped to the appropriate reference genome (mm10 for mouse eyecups) using HISAT2. Gene-level expression counts were quantified using featureCounts. Differential expression analysis was performed using DESeq2, and genes with an adjusted *p*-value < 0.05 and |fold change| >1.5 were considered differentially expressed.

### Gene set enrichment analysis (GSEA)

Gene Set Enrichment Analysis (GSEA) was conducted using the GSEA software (v4.2.3) [66]. Differentially expressed genes identified by DESeq2 were ranked using the signal-to-noise ratio rank metric. Enrichment analysis was performed using predefined gene sets from databases such as Gene Ontology (GO), the Molecular Signatures Database hallmark gene set, Reactome, and Wikipathways [67–70]. Statistical significance of enrichment was determined using a weighted (*p* = 1) scoring calculation scheme with 1000 permutations. Gene sets with sizes larger than 500 and smaller than 5 were excluded from the analysis.

### Joint pathway analysis of lipidomics and transcriptomics

To identify pathways jointly altered at the transcriptional and lipidomic levels, we performed integrated pathway analysis using MetaboAnalyst 6.0 [25]. Differentially expressed genes from bulk RNA sequencing *(p < 0.05)* and significantly altered lipid species from global lipidomics (*p < 0.05*) were uploaded to the MetaboAnalyst joint pathway analysis module. KEGG identifiers were used to map both datasets to known metabolic and signaling pathways. The analysis applied the hypergeometric test for overrepresentation of genes and metabolites, combined with relative-betweenness centrality for pathway topology analysis, producing a combined pathway impact score. Pathways with a *p < 0.05* and impact scores > 0.1 were considered significantly enriched.

### Ethics statement

All animal procedures were approved by the Institutional Animal Care and Use Committee of UC Irvine and conducted in accordance with NIH guidelines.

### Mouse eyecup flatmount dissection and morphology analysis

Mouse eyes were enucleated following euthanasia, and the anterior segment, lens, and neural retina were carefully removed to isolate the posterior eyecup containing the RPE-choroid-sclera complex. Four radial incisions were made to flatten the eyecup, which was then fixed in 4% PFA for 20 min at RT. Eyecups were then washed with PBS and blocked in 2% BSA, 0.1% Triton X-100 for 1 h at room temperature (RT). Samples were incubated overnight at 4 °C with a primary antibody against ZO-1 (rabbit, Invitrogen 61–7300, 1:100). Following washes with PBS, samples were incubated with a fluorescently-labeled secondary antibody. Nuclei were counterstained with Hoechst 33342 (Thermo), and sections were mounted using ProLong Gold Antifade (Thermo). Images were acquired on a Zeiss LSM-900 microscope with Airyscan 2 using 20× magnification. Each flatmount was subdivided into central, mid-peripheral, and peripheral regions based on radial distance from the optic nerve head. Quantitative morphometric analysis of RPE cell shape parameters was performed using the REShAPE pipeline as published [71].

### Two-photon excitation

TPE imaging and fluorescence lifetime data acquisition in mice were done as previously described [72]. The imaging instrument was based on the Leica TCS SP8 Falcon architecture. A custom, tunable light source consisting of a Ti:sapphire laser (Vision S, Coherent) and a pulse selection system was used to generate 780 nm, 4–80 MHz pulsing IR light. A custom periscope objective was used to measure fluorescence from distinct depths in the retina, namely sub-retinal space at the apical side of the RPE and inner retina. During imaging, an anesthetized mouse was surrounded by a heated pad and placed on a mechanical stage with its eye covered with GenTeal gel and a thin 3.2 mm diameter, 0 diopter contact lens (Cantor and Nissel). Fluorescence was measured as pixel mean gray value. Comparison of retinal fluorophores between mouse models was based on spectral detection with channels set to 400–660 nm to measure total fluorescence, 400–550 nm to measure fluorescence from retinyl esters, and 580–680 detecting fluorescence from retinal condensation products. Arbitrary color scale for FLIM imaging was assigned based on the phasor approach [72], briefly phasor FLIM analyses start by displaying the fluorescence lifetime data from each imaging pixel as a point on the 2D graph, and then assigning arbitrary color to selected clusters of phasor points. These procedures were followed by the interpretation of the corresponding phasor FLIM image. FLIM analyses were performed using Leica LAS X FLIM Version 3.5.5 software.

### Transmission electron microscopy

Enucleated eyes were fixed overnight at 4 °C by immersion in 2% paraformaldehyde, 2.5% glutaraldehyde, and 5% CaCl_2_ in 0.1 M cacodylate buffer. After removing the anterior segment under a dissecting microscope, eyecups were processed for Epon embedding as previously described [73]. Ultra-thin sections (~85 nm) were cut using a diamond knife, stained with uranyl acetate and lead citrate, and examined using a Tecnai G2 Spirit BioTWIN transmission electron microscope operated at 60 kV.

### Lipidomic analysis

#### Lipid extraction

Lipid extractions were performed according to the methodology of Bligh and Dyer [74]. In brief, the tissue was homogenized in 200 μL water, transferred to a glass vial, and 750 μL 1:2 (v/v) CHCl_3_: MeOH was added and vortexed. Then 250 μL CHCl_3_ was added and vortexed. Finally, 250 μL ddH_2_O was added and vortexed. The samples were centrifuged at 3000 rpm for 5 min at 4 °C. The lower phase was transferred to a new glass vial, dried under nitrogen, and stored at −20 °C until subsequent lipid analysis.

#### LC-MS/MS

Separation of lipids was performed on an Accucore C30 column (2.6 μm, 2.1 mm × 150 mm, Thermo Scientific). The Q Exactive MS was operated in a full MS scan mode (resolution 70,000 at m/z 200) followed by ddMS2 (17,500 resolution) in both positive and negative modes. The AGC target value was set at 1E6 and 1E5 for the MS and MS/MS scans, respectively. The maximum injection time was 200 ms for MS and 50 ms for MS/MS. HCD was performed with a stepped collision energy of 30 ± 10% for negative and 25% and 30% for positive ion mode with an isolation window of 1.5 Da.

#### Data analysis and post-processing

Data were analyzed with LipidSearch 4.2.21 software. Only peaks with molecular identification grade: A or B were accepted (A: lipid class and fatty acids completely identified or B: lipid class and some fatty acids identified). The relative abundance of each lipid species was obtained by normalization to the total lipid intensity. Significantly changed lipid species (FC > 1.5. *p* < 0.05) were submitted to Lipid Ontology (LION) for lipid ontology analysis. Data visualization was performed using Prism 7 software (GraphPad Software, Inc.).

#### FA analysis

Separation of PUFAs was achieved on an Acquity UPLC® BEH C18 column (1.7 μm, 2.1 mm × 100 mm, Waters Corporation). The Q Exactive MS was operated in a full MS scan mode (resolution 70,000 at m/z 200) in negative mode. For the compounds of interest, a scan range of m/z 250–800 was chosen. The identification of fatty acids was based on retention time and formula.

### Plasma membrane fractionation

Plasma membrane-enriched fractions were isolated from ARPE-19 cells grown in 15 cm dishes using the Minute™ Plasma Membrane/Protein Isolation and Cell Fractionation Kit (Invent Biotechnologies, cat. SM-005) according to the manufacturer’s instructions. Briefly, cells were trypsinized and pelleted by centrifugation at 500 ×*g* for 5 min at 4 °C. Cell pellets were resuspended in Buffer A supplemented with protease inhibitors and homogenized using the provided filter cartridge system. Sequential centrifugation steps were performed to separate cytosolic, organelle, and plasma membrane fractions. The final plasma membrane pellet was resuspended in PBS. Fraction purity was verified by immunoblotting using Na^+^/K^+^ -ATPase as a plasma membrane marker and α-tubulin, PCNA, and TOM20 as cytosolic, nuclear, and mitochondrial/organelle markers, respectively. Plasma membrane fractions were subsequently used for global lipidomic analysis and subjected to C-Laurdan fluorescence spectroscopy to assess membrane order.

### Immunoblotting

Cells were harvested and lysed in RIPA buffer (50 mM Tris-HCl pH 7.4, 150 mM NaCl, 1% NP-40, 0.5% sodium deoxycholate, 0.1% SDS) supplemented with protease and phosphatase inhibitor cocktails (Roche). Protein concentrations were determined using the BCA assay (Thermo Fisher). Equal amounts of protein (5 µg) were mixed with 4× Laemmli sample buffer containing 10% β-mercaptoethanol, boiled at 95 °C for 5 min, and separated by SDS-PAGE on 4–12% gradient gels (Invitrogen). Proteins were transferred to PVDF membranes (Millipore) using the eBlot™ rapid transfer system (Genscript). Membranes were briefly stained with Ponceau S to verify uniform protein transfer and total protein loading before blocking and antibody incubation.

Membranes were blocked in 5% non-fat dry milk in PBS-T (20 mM Tris-HCl pH 7.6, 150 mM NaCl, 0.1% Tween-20) for 1 h at room temperature and incubated overnight at 4 °C with primary antibodies diluted in blocking buffer. Following three washes with PBS-T, membranes were incubated with HRP-conjugated secondary antibodies for 1 h at room temperature. Protein bands were visualized using enhanced chemiluminescence (ECL, Thermo Fisher) and imaged on a ChemiDoc system (Bio-Rad). Densitometric quantification was performed using ImageJ.

### C-Laurdan spectroscopy

Plasma membrane fractions were obtained from ARPE-19 cells grown on 15 cm plates and analyzed as previously described [75]. Briefly, samples were stained with 1 μm C-Laurdan (Tocris Bioscience) through addition of a 1 mM stock solution in DMSO. Samples were incubated with the dye for 30 min on ice. Fluorescence emission spectra were acquired on a Cary Eclipse Fluorescence spectrometer equipped a Peltier temperature controller maintained at 37 °C. Samples were excited at 355 nm with a 10 nm slit width and fluorescence spectra were acquired with a 1.5 nm slit width.

### Quantitative real-time PCR (qPCR)

Total RNA was extracted from differentiated ARPE-19 cells and iPSC-RPE using the RNeasy Plus Mini Kit (Qiagen) following the manufacturer’s instructions. Complementary DNA (cDNA) was synthesized from total RNA using the SuperScript™ VILO cDNA Synthesis Kit (Thermo Fisher Scientific) following the manufacturer’s protocol. Quantitative PCR was performed using iTaq™ Universal SYBR® Green Supermix (Bio-Rad) on a CFX384 Real-Time PCR Detection System (Bio-Rad). Reactions were run in technical triplicates under standard cycling conditions. Relative gene expression was calculated using the ΔΔCt method with normalization to *TBP* as the internal control. Primer sequences for target genes are listed in Table S2.

### Immunofluorescence

Cells were seeded in 12-well dishes containing laminin-coated coverslips and left to adhere overnight. After 3 days (ARPE-19) or 6 days (differentiated ARPE-19) in serum-depleted medium, cells were washed with PBS to remove the medium and fixed with 4% PFA for 15 min at RT. Next, cells were washed with PBS and permeabilized with 0.1% Triton X-100 for 10 min on ice. Cells were then washed with PBS and blocked with 5% BSA for 1 h at RT to minimize nonspecific binding, followed by incubation with primary antibodies in 5% BSA overnight at 4 °C. Following three washes with PBS, cells were incubated with fluorescently-labeled secondary antibodies diluted in 5% BSA for 1 h at RT. Following three washes with PBS, nuclei were counterstained with Hoechst 33342 (Thermo), and coverslips were mounted using ProLong Gold Antifade (Thermo). Images were acquired on a Zeiss LSM-900 microscope with Airyscan 2 using 20× and 40× water immersion. The same brightness/contrast profile was applied to all images within the same experiment. ImageJ imaging software was used for image analysis.

### Flow cytometry

Flow cytometry analyses were performed using a WOLF® G2 cell sorter (NanoCellect Biomedical). Cells were harvested by gentle dissociation, washed in PBS, and resuspended in PBS sorting buffer (Ca^2 +^ - and Mg^2 +^ -free PBS supplemented with 1% BSA, 1 mM EDTA, and 25 mM HEPES (pH 7.0)).

For surface staining, non-permeabilized cells were first blocked in flow cytometry buffer containing 5% BSA to reduce nonspecific binding, followed by incubation with a primary antibody against LAMP2 (1:100) for 30 min at RT. Cells were washed and then incubated with a fluorophore-conjugated secondary antibody for 30 min at RT. After additional washes, cells were resuspended in sorting buffer and analyze by flow cytometry.

Phagocytosis assays were performed following a previously published protocol [76]. Briefly, cells were incubated with FITC-labeled POS (InVision BioResources) for 0.5, 2, and 5 hours at 37 °C. Cells were then rinsed with PBS and incubated with 0.05% trypsin-EDTA for 7 minutes to detach cells and release bound POS. Samples were resuspended in sorting buffer and analyzed by flow cytometry.

### Live-cell calcium imaging

Intracellular calcium dynamics were monitored using the genetically encoded calcium indicator *Salsa6f*- a GCaMP6f-TdTomato fusion protein [77, 78]. Differentiated control and *ELOVL2*^KD^ ARPE-19 cells were plated on 8-well chamber slides (Thermo Scientific 155409) at a density of 0.05 × 10^6^ cells/dish for 6 days and transfected with Salsa6f using TransiT-X2 reagent (Mirus Bio) 24 hours prior to imaging. Cells were imaged in 2 mM Ca^2+^ Ringer solution containing 155 mM NaCl, 4.5 mM KCl, 2 mM CaCl2, 0.5 mM MgCl2, 15 mM glucose, and 15 mM HEPES (pH adjusted to 7.4 with NaOH), and in Ca^2+^ free Ringer solution (similar in composition to 2 mM Ca^2+^ Ringer solution but with 1.5 mM MgCl_2_, 1 mM EGTA and without any Ca^2+^. Imaging was performed on the THUNDER widefield fluorescence microscope (Leica Microsystems equipped with a 20x air objective (HC PL APO 20x/0.80 DRY), Adaptive Focus Control (AFC) to compensate for z-drift, an air-cooled monochrome fluorescence sCMOS camera (Leica K8), and Uno stage top incubator to maintain cell temperature at 37 °C (Okolab). An LED light source in combination with a multi-band filter set (DFT51010 Quad cube, Leica) and external filter wheel (EFW: BP535/70 and BP590/50) was used to sequentially collect GCaMP6f (Ex: 462-496 nm, 12% illumination, Em: 506-532 nm, 150 ms exposure) and TdTomato signal (Ex: 542-566 nm, 2% illumination, Em: 578-610 nm, 100 ms exposure). Images (12-bit, 2048 × 2048 pixels, no binning, 0.325 µm pixel size) were acquired using the Las X software at 1 second interval in a single z-plane.

#### Image analysis

XYT time-lapse videos (.LIF files) were exported to ImageJ, converted to tiff files (16-bit), and background-subtracted (both Green and Red channels). Single-cell analysis was performed by drawing regions of interest (ROIs) around individual cells in the field. Average intensities in the green and red channels were calculated for each ROI at each time point. GCaMP6f/TdTomato [green/red (G/R) ratio] was then obtained to further generate traces showing single-cell and average changes in cytosolic Ca^2+^ over time. Cumulative Ca^2+^ signal was calculated as area under the curve (AUC) using the trapezoid method. Baseline single cell Ca^2+^ level was determined by averaging the lowest 10 G/R ratio values.

### Real-time Annexin V apoptosis monitoring

Phosphatidylserine (PS) exposure and apoptotic activation in live differentiated ARPE-19 cells were monitored using the RealTime-Glo™ Annexin V Apoptosis and Necrosis Assay (Promega, cat. JA1011), according to the manufacturer’s instructions. Briefly, differentiated ARPE-19 cells were transfected with either scrambled control or *ELOVL2*-targeting siRNA and seeded in 96-well plates. After 6 days of maintenance in serum-depleted, B27-supplemented medium, the RealTime-Glo™ Detection Reagent was prepared and added directly to the culture medium. Luminescence and fluorescence signals were measured every hour for 29 hours using a plate reader. H₂O₂-treated control cells (500 µM) were included as a positive control for apoptosis. Data were normalized to baseline luminescence and fluorescence (t = 0) and plotted.

### Endpoint fluorescent Annexin V assay

To validate real-time measurements of PS externalization, an endpoint fluorescent Annexin V assay was performed in parallel. Differentiated ARPE-19 (control and *ELOVL2*^KD^) cells were incubated with Invitrogen™ Annexin V Conjugates for Apoptosis Detection (Alexa Fluor 555) according to the manufacturer’s instructions. Cells were imaged using a Zeiss LSM-900 microscope with Airyscan 2 using 20× magnification and Annexin V puncta were quantified using ImageJ. Data were normalized by applying the same brightness/contrast profile and threshold values. ImageJ’s Analyze Particles feature was used to quantify Annexin V spots over > 6 fields of view for each condition (*n* > 10 cells per field). H₂O₂-treated (1 mM) cells were included as a positive control for apoptosis.

### Lysosome distribution and positioning analysis

For quantitative analysis of lysosome positioning, we used the radial distribution method as previously described [79]. The distance from the cell nucleus to each LAMP1-labeled lysosome was calculated using ImageJ with a radial profiling plugin. Cells were subdivided into concentric zones from the nucleus to the cell periphery (perinuclear 0-30% radius, peripheral 30-60%). The proportion of lysosomes within each zone was calculated and compared between wild-type and *ELOVL2*^KD^ cells. For quantitative analysis of lysosome positioning, we used the radial distribution method as previously described [79]. The distance from the cell nucleus to each LAMP1-labelled lysosome was calculated using ImageJ with a radial profiling plugin.

### Proximity ligation assay

Reagents for the PLA of differentiated ARPE-19 cells were purchased and used using instructions from Sigma-Aldrich (DUO92004 and DUO92002). Briefly, cells were fixed on coverslips and prepared for staining with primary antibodies as described above. Anti-ZO-1, anti-LAMP2, and anti-IgG were used at 1:100 dilution. Anti-IgG was used as a negative control. Instead of fluorescently tagged secondary antibodies, specimens were incubated with anti-mouse and anti-rabbit oligonucleotide-tethered secondary antibodies. Close proximity of target antigens allows their respective secondary antibody nucleotide sequences to hybridize. Following a 30-min 37 °C incubation with ligase and additional oligonucleotides, a closed DNA circle was formed. A subsequent step involving polymerase-driven rolling circle amplification incorporated fluorescently labeled nucleotides. Fluorescent nucleotides used in this experiment fluoresce in the red channel upon excitation. Fluorescent spots appear at sites of ZO-1/LAMP2 protein-protein interactions when using primary antibodies and were evaluated using confocal microscopy and quantified using ImageJ. Data were normalized by applying the same brightness/contrast profile and threshold values. ImageJ’s Analyze Particles feature was used to quantify PLA spots over 10 fields of view for each condition (*n* > 30). Raw values were then converted to fold change with respect to the appropriate control.

### β-hexosaminidase release assay

Extracellular release of β-hexosaminidase was quantified as a marker of lysosomal exocytosis. Cells were plated in 12-well plates and cultured in phenol-red free media for 6 days. Culture supernatants were collected and centrifuged at 300 x*g* for 10 min and 2000 x*g* for 10 min to remove cell debris. The clarified supernatants were then concentrated using Amicon Ultra centrifugal filters (50 kDa cutoff at 3,000 x*g* for 5 min, followed by 3 kDa cutoff at 14,000 x*g* for 30 min). Cells were then washed once with ice-cold PBS and lysed in RIPA buffer supplemented with protease inhibitor cocktail. Both supernatants and lysates were incubated with 4-methylumbelliferyl N-acetyl-β-D-glucosaminide substrate (2 mM in sodium-citrate-phosphate buffer, pH 4.5) for 30 min at RT. The reaction was terminated by adding 0.2 M glycine, pH 10. Fluorescence intensity was measured at excitation/emission wavelengths of 365/450 nm using a microplate reader. Enzyme release was quantified as the ratio of β-hexosaminidase activity in the supernatant to that in the corresponding lysate, providing a measure of relative extracellular enzyme secretion.

### Lattice light-sheet microscopy of live cells

Four-dimensional imaging was performed using a custom-built lattice light-sheet microscope developed by the Eric Betzig laboratory at HHMI Janelia Research Campus and UC Berkeley [80]. The system was equipped with a 0.6 NA excitation objective (Thorlabs TL20X-MPL) and a 1.0 NA detection objective (Zeiss W Plan-Apochromat 20×/1.0, model #421452-9800) and a Hamamatsu ORCA-Fusion BT sCMOS camera.

For live-cell labeling, cells were incubated with 50 nM LysoTracker Green for 30 min at 37 °C. Following LysoTracker labeling, cells were washed and incubated with 5 µg/mL CellMask Deep Red plasma membrane stain for 5 min at 37 °C. After staining, cells were washed and imaged immediately in phenol red-free imaging medium.

Excitation was provided by 488-nm and 642-nm lasers to image LysoTracker Green and CellMask Deep Red, respectively. Illumination was generated using a multiple Bessel beam in a lattice light-sheet pattern with a numerical aperture range of NA_min_ = 0.35 to NA_max_ = 0.40. During imaging, samples were maintained at 37 °C in a humidified atmosphere containing 5% CO_2_. Raw lattice light-sheet microscopy data were preprocessed using the LiveLattice pipeline [31], including camera background subtraction, photobleaching correction, sample-scan deconvolution, deskewing, and rotation into a standard Cartesian coordinate system. Each preprocessed 3D volume comprised 864 × 1536 × 202 voxels (x, y, z) with an isotropic voxel size of 0.111 µm. Time-lapse imaging was performed at 5.6-s intervals for a total of 60 frames.

### Lipidomics-based phagocytosis assay

Phagocytosis of POS by iPSC-RPE was assessed using a lipidomics-based approach following a previously published protocol [32]. Briefly, purified bovine POS (InVision BioResources) were added to confluent iPSC-RPE monolayers at a ratio of 10 or 20 POS per cell and incubated for 3 hours at 37 °C. After incubation, cells were washed extensively with phosphate-buffered saline (PBS) to remove unbound POS, dissociated by trypsinization, and collected by centrifugation. Cell pellets were then subjected to lipid extraction and hydrolysis for downstream lipidomic analysis. VLC-PUFAs 32:6, 34:4, 34:5, 34:6, and 36:6 were measured as markers of internalized POS.

### Intravitreal injections

Intravitreal injections were performed as previously described [12] as part of a separate study. For the present work, RPE eyecups from these animals were collected and analyzed to assess lipidomic and cellular outcomes.

## Electronic supplementary material

Below is the link to the electronic supplementary material.

Supplementary Material 1

Supplementary Material 2

## Data Availability

All data supporting the findings of this study are available in the main text, extended data figures and supplementary materials. Bulk RNA sequencing data generated in this study have been deposited in the NCBI Gene Expression Omnibus (GEO) under accession number GSE343357. The MS lipidomic data have been deposited in Dryad (DOI https://doi.org/10.5061/dryad.34tmpg51p). Elovl2C234W *Elovl2*^*C234W*^ animals are available to interested researchers through a material transfer agreement with University of California, Irvine.

## References

[1] Lakkaraju A, Umapathy A, Tan LX, Daniele L, Philp NJ, Boesze-Battaglia K, et al. The cell biology of the retinal pigment epithelium. Prog Retin Eye Res. 2020;78:100846. 10.1016/j.preteyeres.2020.100846. PMC8941496 32105772

[2] Handa JT, Bowes Rickman C, Dick AD, Gorin MB, Miller JW, Toth CA, et al. A systems biology approach towards understanding and treating non-neovascular age-related macular degeneration. Nat Commun. 2019;10(1):3347. 10.1038/s41467-019-11262-1. 31350409 PMC6659646

[3] Tan LX, Germer CJ, La Cunza N, Lakkaraju A. Complement activation, lipid metabolism, and mitochondrial injury: converging pathways in age-related macular degeneration. Redox Biol. 2020;37:101781. 10.1016/j.redox.2020.101781. 33162377 PMC7767764

[4] Sunshine H, Iruela-Arispe ML. Membrane lipids and cell signaling. Curr Opin Lipidol. 2017;28(5):408–13. 10.1097/MOL.0000000000000443. 28692598 PMC5776726

[5] Harayama T, Riezman H. Understanding the diversity of membrane lipid composition. Nat Rev Mol Cell Biol. 2018;19(5):281–96. 10.1038/nrm.2017.138. 29410529

[6] Datta S, Cano M, Ebrahimi K, Wang L, Handa JT. The impact of oxidative stress and inflammation on RPE degeneration in non-neovascular AMD. Prog Retin Eye Res. 2017;60:201–18. 10.1016/j.preteyeres.2017.03.002. 28336424 PMC5600827

[7] Cheng V, Rallabandi R, Gorusupudi A, Lucas S, Rognon G, Bernstein PS, et al. Influence of very-long-chain polyunsaturated fatty acids on membrane structure and dynamics. Biophys J. 2022;121(14):2730–41. 10.1016/j.bpj.2022.06.015. 35711144 PMC9382336

[8] Garagnani P, Bacalini MG, Pirazzini C, Gori D, Giuliani C, Mari D, et al. Methylation of ELOVL2 gene as a new epigenetic marker of age. Aging Cell. 2012;11(6):1132–34. 10.1111/acel.12005. 23061750

[9] Spólnicka M, Pośpiech E, Pepłońska B, Zbieć-Piekarska R, Makowska Ż, Pięta A, et al. DNA methylation in ELOVL2 and C1orf132 correctly predicted chronological age of individuals from three disease groups. Int J Legal Med. 2018;132(1):1–11. 10.1007/s00414-017-1636-0. 28725932 PMC5748441

[10] Bacalini MG, Deelen J, Pirazzini C, De Cecco M, Giuliani C, Lanzarini C, et al. Systemic age-associated DNA hypermethylation of ELOVL2 gene: in vivo and in vitro evidences of a cell replication process. J Gerontol Biol Sci Med Sci. 2017;72(8):1015–23. 10.1093/gerona/glw185. PMC5861890 27672102

[11] Chen D, Chao DL, Rocha L, Kolar M, Nguyen Huu VA, Krawczyk M, et al. The lipid elongation enzyme ELOVL2 is a molecular regulator of aging in the retina. Aging Cell. 2020;19(2):e13100. 10.1111/acel.13100. PMC6996962 31943697

[12] Gao F, Tom E, Rydz C, Cho W, Kolesnikov AV, Sha Y, et al. Retinal polyunsaturated fatty acid supplementation reverses aging-related vision decline in mice. Sci Transl Med. 2025;17(817):eads5769. 10.1126/scitranslmed.ads5769. PMC13033240 40991728

[13] Zhao X, et al. Lipidomic profiling reveals age-dependent changes in complex plasma membrane lipids that regulate neural stem cell aging. 2022.08.18.503095 [Preprint]. 2022. 10.1101/2022.08.18.503095. PMC13418745 42525742

[14] Mota-Martorell N, Andrés-Benito P, Martín-Gari M, Galo-Licona JD, Sol J, Fernández-Bernal A, et al. Selective brain regional changes in lipid profile with human aging. GeroScience. 2022;44(2):763–83. 10.1007/s11357-022-00527-1. 35149960 PMC9135931

[15] Dai Y, Tang H, Pang S. The crucial roles of phospholipids in aging and lifespan regulation. Front Physiol. 2021;12:775648. 10.3389/fphys.2021.775648. 34887779 PMC8650052

[16] Skowronska-Krawczyk D, Budin I. Aging membranes: unexplored functions for lipids in the lifespan of the central nervous system. Exp Gerontol. 2020;131:110817. 10.1016/j.exger.2019.110817. 31862420 PMC7877915

[17] Butler JM, Supharattanasitthi W, Yang YC, Paraoan L. RNA-seq analysis of ageing human retinal pigment epithelium: unexpected up-regulation of visual cycle gene transcription. J Cell Mol Med. 2021;25(12):5572–85. 10.1111/jcmm.16569. 33934486 PMC8184696

[18] Thomas PD, Ebert D, Muruganujan A, Mushayahama T, Albou L-P, Mi H. PANTHER: making genome-scale phylogenetics accessible to all. Protein Sci. 2022;31(1):8–22. 10.1002/pro.4218. 34717010 PMC8740835

[19] Geerlings MJ, de Jong EK, den Hollander AI. The complement system in age-related macular degeneration: a review of rare genetic variants and implications for personalized treatment. Mol Immunol. 2017;84:65–76. 10.1016/j.molimm.2016.11.016. 27939104 PMC5380947

[20] Armento A, Ueffing M, Clark SJ. The complement system in age-related macular degeneration. Cell Mol Life Sci. 2021;78(10):4487–505. 10.1007/s00018-021-03796-9. 33751148 PMC8195907

[21] Wilke GA, Apte RS. Complement regulation in the eye: implications for age-related macular degeneration. J Clin Invest. 2024;134(9). 10.1172/JCI178296. PMC11060743 38690727

[22] Kim Y-K, Yu H, Summers VR, Donaldson KJ, Ferdous S, Shelton D, et al. Morphometric analysis of retinal pigment epithelial cells from C57BL/6J mice during aging. Invest Ophthalmol Vis Sci. 2021;62(2):32. 10.1167/iovs.62.2.32. 33616620 PMC7910641

[23] Pilgrim MG, Lengyel I, Lanzirotti A, Newville M, Fearn S, Emri E, et al. Subretinal pigment epithelial deposition of drusen components including hydroxyapatite in a primary cell culture model. Invest Ophthalmol Vis Sci. 2017;58(2):708–19. 10.1167/iovs.16-21060. 28146236 PMC5295770

[24] Chen S, Abu-Qamar O, Kar D, Messinger JD, Hwang Y, Moult EM, et al. Ultrahigh resolution OCT markers of normal aging and early age-related macular degeneration. Ophthalmol Sci. 2023;3(3):100277. 10.1016/j.xops.2023.100277. 36970115 PMC10034509

[25] Pang Z, Lu Y, Zhou G, Hui F, Xu L, Viau C, et al. MetaboAnalyst 6.0: towards a unified platform for metabolomics data processing, analysis and interpretation. Nucleic Acids Res. 2024;52(W1):W398–406. 10.1093/nar/gkae253. PMC11223798 38587201

[26] Hazim RA, Volland S, Yen A, Burgess BL, Williams DS. Rapid differentiation of the human RPE cell line, ARPE-19, induced by nicotinamide. Exp Eye Res. 2019;179:18–24. 10.1016/j.exer.2018.10.009. 30336127 PMC6360117

[27] Levitan I. Evaluating membrane structure by Laurdan imaging: disruption of lipid packing by oxidized lipids. Curr Top Membr. 2021;88:235–56. 34862028 10.1016/bs.ctm.2021.10.003 PMC8929669

[28] Reddy A, Caler EV, Andrews NW. Plasma membrane repair is mediated by Ca2+-regulated exocytosis of lysosomes. Cell. 2001;106(2):157–69. 10.1016/S0092-8674(01)00421-4. 11511344

[29] Encarnação M, Espada L, Escrevente C, Mateus D, Ramalho J, Michelet X, et al. A Rab3a-dependent complex essential for lysosome positioning and plasma membrane repair. J Cell Biol. 2016;213(6):631–40. 10.1083/jcb.201511093. 27325790 PMC4915190

[30] Corrotte M, Castro-Gomes T. Lysosomes and plasma membrane repair. Curr Top Membr. 2019;84:1–16. 31610859 10.1016/bs.ctm.2019.08.001

[31] Wang Z, Hakozaki H, McMahon G, Medina-Carbonero M, Schöneberg J. LiveLattice: real-time visualisation of tilted light-sheet microscopy data using a memory-efficient transformation algorithm. J Microscopy. 2025;297(2):123–34. 10.1111/jmi.13358. PMC11733850 39360400

[32] Gao F, Tom E, Lieffrig SA, Finnemann SC, Skowronska-Krawczyk D. A novel quantification method for retinal pigment epithelium phagocytosis using a very-long-chain polyunsaturated fatty acids-based strategy. Front Mol Neurosci. 2023;16. 10.3389/fnmol.2023.1279457. PMC10622967 37928068

[33] Klionsky DJ, Abdel-Aziz AK, Abdelfatah S, Abdellatif M, Abdoli A, Abel S, et al. Guidelines for the use and interpretation of assays for monitoring autophagy (4th edition)1. Autophagy. 2021;17(1):1–382. 10.1080/15548627.2020.1797280. 33634751 PMC7996087

[34] Sangiovanni JP, Agrón E, Meleth AD, Reed GF, Sperduto RD, Clemons TE, et al. ω–3 long-chain polyunsaturated fatty acid intake and 12-y incidence of neovascular age-related macular degeneration and central geographic atrophy: AREDS report 30, a prospective cohort study from the Age-Related Eye Disease Study. Am J Clin Nutr. 2009;90(6):1601–07. 10.3945/ajcn.2009.27594. 19812176 PMC2777471

[35] van Leeuwen EM, Emri E, Merle BMJ, Colijn JM, Kersten E, Cougnard-Gregoire A, et al. A new perspective on lipid research in age-related macular degeneration. Prog Retin Eye Res. 2018;67:56–86. 10.1016/j.preteyeres.2018.04.006. 29729972

[36] Liu A, Chang J, Lin Y, Shen Z, Bernstein PS. Long-chain and very long-chain polyunsaturated fatty acids in ocular aging and age-related macular degeneration. J Lipid Res. 2010;51(11):3217–29. 10.1194/jlr.M007518. 20688753 PMC2952562

[37] Kaur G, Tan LX, Rathnasamy G, La Cunza N, Germer CJ, Toops KA, et al. Aberrant early endosome biogenesis mediates complement activation in the retinal pigment epithelium in models of macular degeneration. Proc Natl Acad Sci USA. 2018;115(36):9014–19. 10.1073/pnas.1805039115. 30126999 PMC6130344

[38] Lewandowski D, Foik AT, Smidak R, Choi EH, Zhang J, Hoang T, et al. Inhibition of ceramide accumulation in AdipoR1–/– mice increases photoreceptor survival and improves vision. JCI Insight. 2022;7(4):e156301. 10.1172/jci.insight.156301. PMC8876453 35015730

[39] Lewandowski D, Gao F, Imanishi S, Tworak A, Bassetto M, Dong Z, et al. Restoring retinal polyunsaturated fatty acid balance and retina function by targeting ceramide in AdipoR1-deficient mice. J Biol Chem. 2024;300(5):107291. 10.1016/j.jbc.2024.107291. 38636661 PMC11107370

[40] Kunchithapautham K, Rohrer B. Sublytic membrane-attack-complex (MAC) activation alters regulated rather than constitutive vascular endothelial growth factor (VEGF) secretion in retinal pigment epithelium monolayers. J Biol Chem. 2011;286(27):23717–24. 10.1074/jbc.M110.214593. 21566137 PMC3129152

[41] Tan LX, Toops KA, Lakkaraju A. Protective responses to sublytic complement in the retinal pigment epithelium. Proc Natl Acad Sci USA. 2016;113(31):8789–94. 10.1073/pnas.1523061113. 27432952 PMC4978296

[42] Platt FM, d’Azzo A, Davidson BL, Neufeld EF, Tifft CJ. Lysosomal storage diseases. Nat Rev Dis Primers. 2018;4(1):27. 10.1038/s41572-018-0025-4. 30275469

[43] Jo DS, Park NY, Cho D-H. Peroxisome quality control and dysregulated lipid metabolism in neurodegenerative diseases. Exp Mol Med. 2020;52(9):1486–95. 10.1038/s12276-020-00503-9. 32917959 PMC8080768

[44] Toops KA, Tan LX, Jiang Z, Radu RA, Lakkaraju A. Cholesterol-mediated activation of acid sphingomyelinase disrupts autophagy in the retinal pigment epithelium. Mol Biol Cell. 2015;26(1):1–14. 10.1091/mbc.e14-05-1028. 25378587 PMC4279221

[45] Cunza NL, Tan LX, Thamban T, Germer CJ, Rathnasamy G, Toops KA, et al. Mitochondria-dependent phase separation of disease-relevant proteins drives pathological features of age-related macular degeneration. JCI Insight. 2021;6(9). 10.1172/jci.insight.142254. PMC8262309 33822768

[46] Zhang KR, Jankowski CSR, Marshall R, Nair R, Más Gómez N, Alnemri A, et al. Oxidative stress induces lysosomal membrane permeabilization and ceramide accumulation in retinal pigment epithelial cells. Dis Model Mech. 2023;16(7):dmm050066. 10.1242/dmm.050066. PMC10399446 37401371

[47] Tam C, Idone V, Devlin C, Fernandes MC, Flannery A, He X, et al. Exocytosis of acid sphingomyelinase by wounded cells promotes endocytosis and plasma membrane repair. J Cell Biol. 2010;189(6):1027–38. 10.1083/jcb.201003053. 20530211 PMC2886342

[48] Andrews NW. Solving the secretory acid sphingomyelinase puzzle: insights from lysosome-mediated parasite invasion and plasma membrane repair. Cell Microbiol. 2019;21(11):e13065. 10.1111/cmi.13065. PMC6842087 31155842

[49] Ramachandran PV, Savini M, Folick AK, Hu K, Masand R, Graham BH, et al. Lysosomal signaling promotes longevity by adjusting mitochondrial activity. Dev Cell. 2019;48(5):685–96.e5. 10.1016/j.devcel.2018.12.022. 30713071 PMC6613828

[50] Savini M, Folick A, Lee Y-T, Jin F, Cuevas A, Tillman MC, et al. Lysosome lipid signalling from the periphery to neurons regulates longevity. Nat Cell Biol. 2022;24(6):906–16. 10.1038/s41556-022-00926-8. 35681008 PMC9203275

[51] Zhang Q, Dang W, Wang MC. Lysosomes signal through the epigenome to regulate longevity across generations. Science. 2025;389(6767):1353–60. 10.1126/science.adn8754. 40997170 PMC12831228

[52] Li TY, Wang Q, Gao AW, Li X, Sun Y, Mottis A, et al. Lysosomes mediate the mitochondrial UPR via mTORC1-dependent ATF4 phosphorylation. Cell Discov. 2023;9(1):92. 10.1038/s41421-023-00589-1. 37679337 PMC10484937

[53] Lawrence RE, Zoncu R. The lysosome as a cellular centre for signalling, metabolism and quality control. Nat Cell Biol. 2019;21(2):133–42. 10.1038/s41556-018-0244-7. 30602725

[54] Shin HR, Citron YR, Wang L, Tribouillard L, Goul CS, Stipp R, et al. Lysosomal GPCR-like protein LYCHOS signals cholesterol sufficiency to mTORC1. Science. 2022;377(6612):1290–98. 10.1126/science.abg6621. 36007018 PMC10023259

[55] Agbaga M-P, Brush RS, Mandal MNA, Henry K, Elliott MH, Anderson RE. Role of stargardt-3 macular dystrophy protein (ELOVL4) in the biosynthesis of very long chain fatty acids. Proc Natl Acad Sci USA. 2008;105(35):12843–48. 10.1073/pnas.0802607105. 18728184 PMC2525561

[56] Esteve-Rudd J, Hazim RA, Diemer T, Paniagua AE, Volland S, Umapathy A, et al. Defective phagosome motility and degradation in cell nonautonomous RPE pathogenesis of a dominant macular degeneration. Proc Natl Acad Sci USA. 2018;115(21):5468–73. 10.1073/pnas.1709211115. 29735674 PMC6003516

[57] Lewandowski D, Sander CL, Tworak A, Gao F, Xu Q, Skowronska-Krawczyk D. Dynamic lipid turnover in photoreceptors and retinal pigment epithelium throughout life. Prog Retin Eye Res. 2022;89:101037. 10.1016/j.preteyeres.2021.101037. 34971765 PMC10361839

[58] SanGiovanni JP, Chew EY. The role of omega-3 long-chain polyunsaturated fatty acids in health and disease of the retina. Prog Retin Eye Res. 2005;24(1):87–138. 10.1016/j.preteyeres.2004.06.002. 15555528

[59] Cell culture in the neurosciences. Boston, MA: Springer US; 1985. 10.1007/978-1-4613-2473-7.

[60] Kuo H-H, Gao X, DeKeyser J-M, Fetterman KA, Pinheiro EA, Weddle CJ, et al. Negligible-cost and weekend-free chemically defined human iPSC culture. Stem Cell Rep. 2020;14(2):256–70. 10.1016/j.stemcr.2019.12.007. PMC7013200 31928950

[61] Brewer GJ, Torricelli JR, Evege EK, Price PJ. Optimized survival of hippocampal neurons in B27-supplemented neurobasal™, a new serum-free medium combination. J Neurosci Res. 1993;35(5):567–76. 10.1002/jnr.490350513. 8377226

[62] Green J, Bhise N, Wahlin K, Zack D. Evaluating the potential of poly(beta-amino ester) nanoparticles for reprogramming human fibroblasts to become induced pluripotent stem cells. Int J Nanomed. 2013;8:4641–58. 10.2147/IJN.S53830. PMC3857166 24348039

[63] Jurlina SL, Jones MK, Agarwal D, De La Toba DV, Kambli N, Su F, et al. A tet-inducible CRISPR platform for high-fidelity editing of human pluripotent stem cells. Genes (Basel). 2022;13(12):2363. 10.3390/genes13122363. 36553630 PMC9777998

[64] Agarwal D, Dash N, Mazo KW, Chopra M, Avila MP, Patel A, et al. Human retinal ganglion cell neurons generated by synchronous BMP inhibition and transcription factor mediated reprogramming. npj Regen Med. 2023;8(1):55. 10.1038/s41536-023-00327-x. 37773257 PMC10541876

[65] Sharma R, Bose D, Montford J, Ortolan D, Bharti K. Triphasic developmentally guided protocol to generate retinal pigment epithelium from induced pluripotent stem cells. Star Protoc. 2022;3(3):101582. 10.1016/j.xpro.2022.101582. 35880133 PMC9307589

[66] Subramanian A, Tamayo P, Mootha VK, Mukherjee S, Ebert BL, Gillette MA, et al. Gene set enrichment analysis: a knowledge-based approach for interpreting genome-wide expression profiles. Proc Natl Acad Sci USA. 2005;102(43):15545–50. 10.1073/pnas.0506580102. 16199517 PMC1239896

[67] Ontology Consortium G, Gene Ontology Consortium. The Gene Ontology (GO) database and informatics resource. Nucleic Acids Res. 2004;32(90001):258D–61. 10.1093/nar/gkh036. PMC308770 14681407

[68] Agrawal A, Balcı H, Hanspers K, Coort SL, Martens M, Slenter DN, et al. WikiPathways 2024: next generation pathway database. Nucleic Acids Res. 2024;52(D1):D679–89. 10.1093/nar/gkad960. PMC10767877 37941138

[69] Milacic M, Beavers D, Conley P, Gong C, Gillespie M, Griss J, et al. The reactome pathway knowledgebase 2024. Nucleic Acids Res. 2024;52(D1):D672–78. 10.1093/nar/gkad1025. PMC10767911 37941124

[70] Liberzon A, Birger C, Thorvaldsdóttir H, Ghandi M, Mesirov J, Tamayo P. The Molecular Signatures Database (MSigDB) hallmark gene set collection. Cell Syst. 2015;1(6):417–25. 10.1016/j.cels.2015.12.004. 26771021 PMC4707969

[71] Ortolan D, Sharma R, Volkov A, Maminishkis A, Hotaling NA, Huryn LA, et al. Single-cell–resolution map of human retinal pigment epithelium helps discover subpopulations with differential disease sensitivity. Proc Natl Acad Sci USA. 2022;119(19):e2117553119. 10.1073/pnas.2117553119. PMC9171647 35522714

[72] Palczewska G, Boguslawski J, Stremplewski P, Kornaszewski L, Zhang J, Dong Z, et al. Noninvasive two-photon optical biopsy of retinal fluorophores. Proc Natl Acad Sci USA. 2020;117(36):22532–43. 10.1073/pnas.2007527117. 32848058 PMC7486747

[73] Bonilha VL, Bell BA, Rayborn ME, Yang X, Kaul C, Grossman GH, et al. Loss of DJ-1 elicits retinal abnormalities, visual dysfunction, and increased oxidative stress in mice. Exp Eye Res. 2015;139:22–36. 10.1016/j.exer.2015.07.014. 26215528 PMC4573318

[74] Bligh EG, Dyer WJ. A rapid method of total lipid extraction and purification. Can J Biochem Physiol. 1959;37(1):911–17. 10.1139/y59-099. 13671378

[75] Wong AM, Budin I. Organelle-targeted laurdans measure heterogeneity in subcellular membranes and their responses to saturated lipid stress. ACS Chem Biol. 2024;19(8):1773–85. 10.1021/acschembio.4c00249. 39069657 PMC11670155

[76] Westenskow PD, Moreno SK, Krohne TU, Kurihara T, Zhu S, Zhang Z-N, et al. Using flow cytometry to compare the dynamics of photoreceptor outer segment phagocytosis in iPS-derived RPE cells. Invest Ophthalmol Vis Sci. 2012;53(10):6282–90. 10.1167/iovs.12-9721. 22871841 PMC3444211

[77] Dong TX, Othy S, Jairaman A, Skupsky J, Zavala A, Parker I, et al. T-cell calcium dynamics visualized in a ratiometric tdTomato-GCaMP6f transgenic reporter mouse. eLife. 2017;6:e32417. 10.7554/eLife.32417. PMC5747524 29239725

[78] Jairaman A, McQuade A, Granzotto A, Kang YJ, Chadarevian JP, Gandhi S, et al. TREM2 regulates purinergic receptor-mediated calcium signaling and motility in human iPSC-derived microglia. eLife. 2022;11:e73021. 10.7554/eLife.73021. PMC8906810 35191835

[79] Williamson CD, Guardia CM, De Pace R, Bonifacino JS, Saric A. Measurement of lysosome positioning by shell analysis and line scan. Methods Mol Biol. 2022;2473:285–306. 35819772 10.1007/978-1-0716-2209-4_19 PMC11072972

[80] Fu T-M, et al. A multimodal adaptive optical microscope for in vivo imaging from molecules to organisms. 2025.06.02.657494 [Preprint]. 2025. 10.1101/2025.06.02.657494. PMC13310421 42174242

