# Supplementary material for: Lipid-driven membrane remodeling engages a lysosome-dependent adaptive repair program during retinal aging
Source: Mol Neurodegener. 2026 Aug 29;21:56. doi: 10.1186/s13024-026-00990-w (PMC13563809; doi:10.1186/s13024-026-00990-w)
Supplement: Supplementary file 1 [file 13024_2026_990_MOESM2_ESM.pdf]

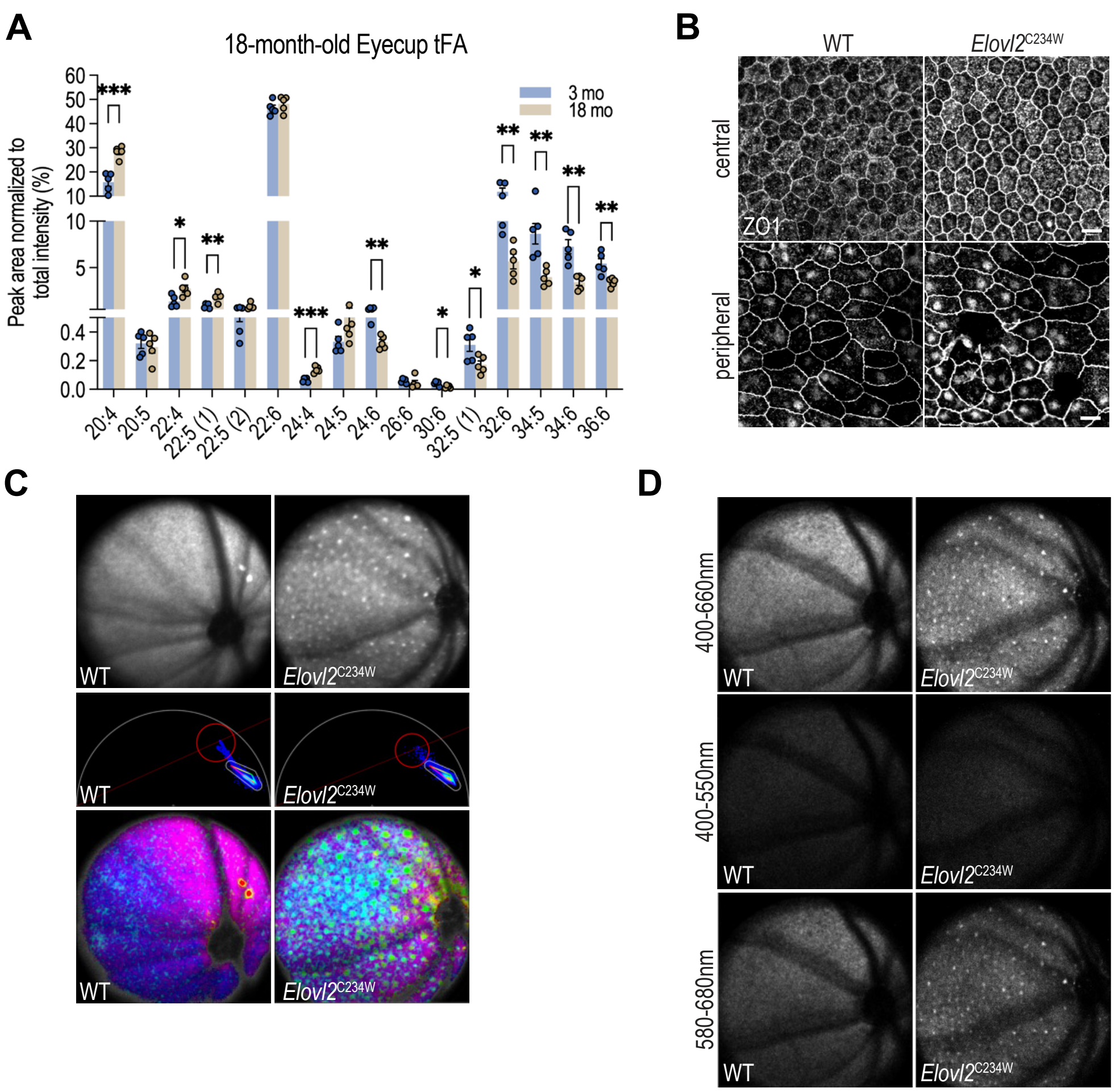
A
18-month-old Eyecup tFA
3 mo
18 mo
Peak area normalized to total intensity (%)
20:4
20:5
22:4
22:5 (1)
22:5 (2)
22:6
24:4
24:5
24:6
26:6
30:6
32:5 (1)
32:6
34:5
34:6
36:6
B
WT
Elovl2C234W
central
peripheral
ZO1
C
WT
Elovl2C234W
D
400-660nm
400-550nm
580-680nm
WT
Elovl2C234W

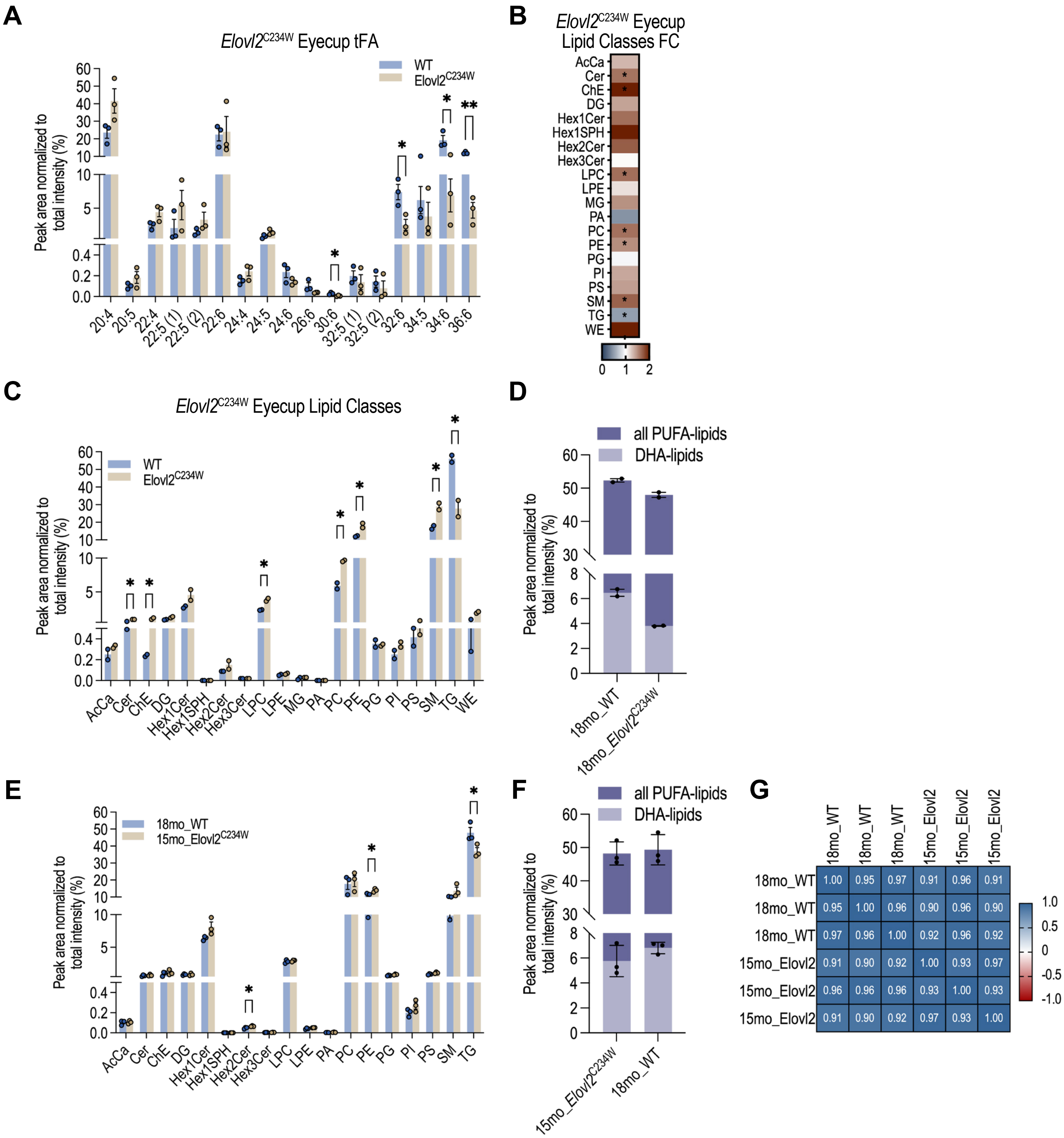

A
Elovl2C234W Eyecup tFA
WT
Elovl2C234W
Peak area normalized to total intensity (%)
20:4
20:5
22:4
22:5 (1)
22:5 (2)
22:6
24:4
24:5
24:6
26:6
30:6
32:5 (1)
32:5 (2)
32:6
34:5
34:6
36:6
B
Elovl2C234W Eyecup Lipid Classes FC
AcCa
Cer
ChE
DG
Hex1Cer
Hex1SPH
Hex2Cer
Hex3Cer
LPC
LPE
MG
PA
PC
PE
PG
PI
PS
SM
TG
WE
C
Elovl2C234W Eyecup Lipid Classes
WT
Elovl2C234W
Peak area normalized to total intensity (%)
D
all PUFA-lipids
DHA-lipids
Peak area normalized to total intensity (%)
18mo_WT
18mo_Elovl2C234W
E
18mo_WT
15mo_Elovl2C234W
Peak area normalized to total intensity (%)
F
all PUFA-lipids
DHA-lipids
Peak area normalized to total intensity (%)
15mo_Elovl2C234W
18mo_WT
G
18mo_WT
18mo_WT
18mo_WT
15mo_Elovl2
15mo_Elovl2
15mo_Elovl2
18mo_WT 1.00 0.95 0.97 0.91 0.96 0.91
18mo_WT 0.95 1.00 0.96 0.90 0.96 0.90
18mo_WT 0.97 0.96 1.00 0.92 0.96 0.92
15mo_Elovl2 0.91 0.90 0.92 1.00 0.93 0.97
15mo_Elovl2 0.96 0.96 0.96 0.93 1.00 0.93
15mo_Elovl2 0.91 0.90 0.92 0.97 0.93 1.00

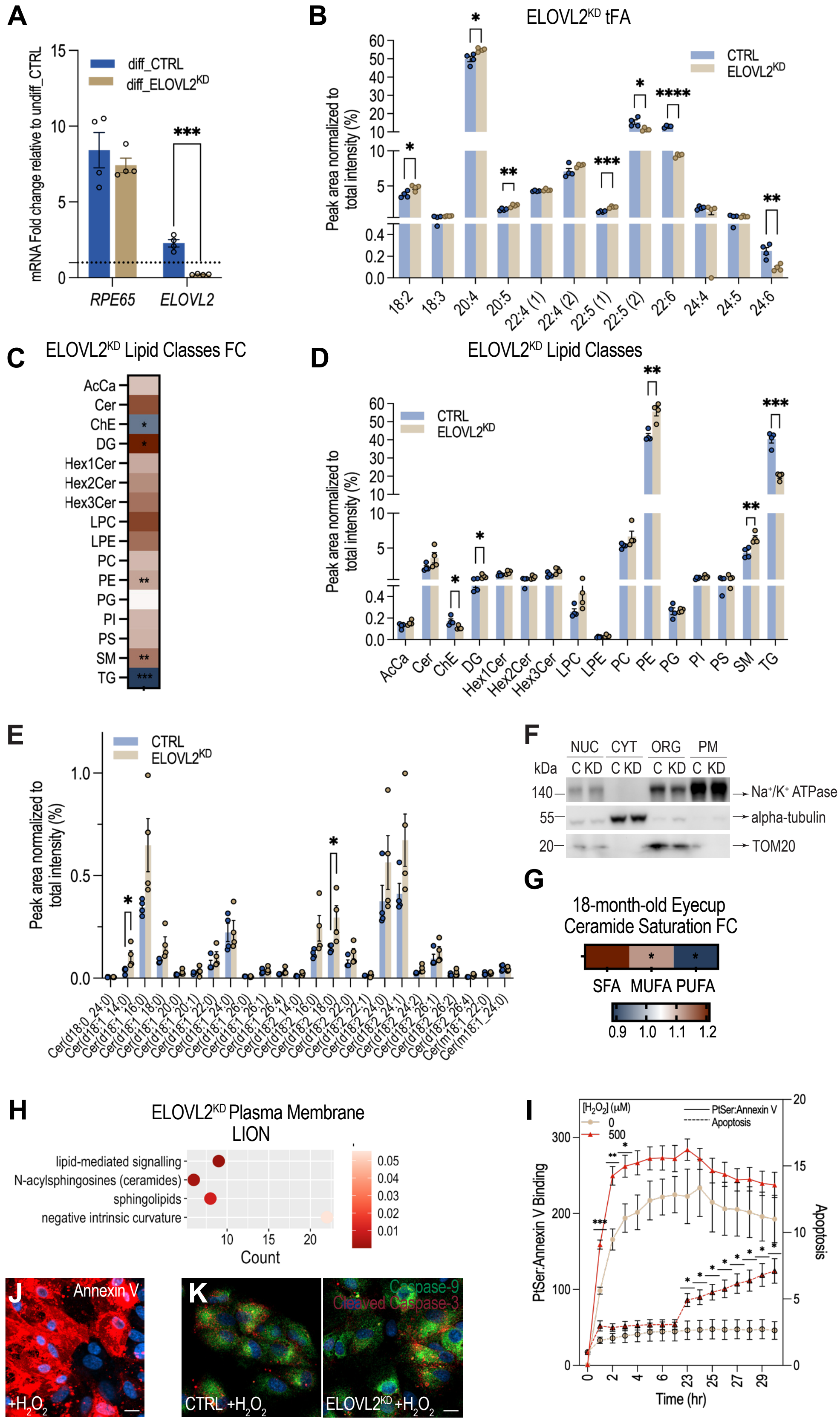

A
diff_CTRL
diff_ELOVL2KD
mRNA Fold change relative to undiff_CTRL
RPE65
ELOVL2
B
ELOVL2KD tFA
CTRL
ELOVL2KD
Peak area normalized to total intensity (%)
18:2
18:3
20:4
20:5
22:4 (1)
22:4 (2)
22:5 (1)
22:5 (2)
22:6
24:4
24:5
24:6
C
ELOVL2KD Lipid Classes FC
AcCa
Cer
ChE
DG
Hex1Cer
Hex2Cer
Hex3Cer
LPC
LPE
PC
PE
PG
PI
PS
SM
TG
D
ELOVL2KD Lipid Classes
CTRL
ELOVL2KD
Peak area normalized to total intensity (%)
E
CTRL
ELOVL2KD
Peak area normalized to total intensity (%)
Cer(d18:0_24:0)
Cer(d18:1_14:0)
Cer(d18:1_16:0)
Cer(d18:1_18:0)
Cer(d18:1_20:0)
Cer(d18:1_20:1)
Cer(d18:1_22:0)
Cer(d18:1_24:0)
Cer(d18:1_26:0)
Cer(d18:1_26:1)
Cer(d18:1_26:4)
Cer(d18:2_14:0)
Cer(d18:2_16:0)
Cer(d18:2_18:0)
Cer(d18:2_22:0)
Cer(d18:2_22:1)
Cer(d18:2_24:0)
Cer(d18:2_24:1)
Cer(d18:2_24:2)
Cer(d18:2_26:1)
Cer(d18:2_26:2)
Cer(d18:2_26:4)
Cer(m18:1_22:0)
Cer(m18:1_24:0)
F
NUC
CYT
ORG
PM
kDa
C KD C KD C KD C KD
140
55
20
Na+/K+ ATPase
alpha-tubulin
TOM20
G
18-month-old Eyecup
Ceramide Saturation FC
SFA
MUFA
PUFA
0.9 1.0 1.1 1.2
H
ELOVL2KD Plasma Membrane
LION
lipid-mediated signalling
N-acylsphingosines (ceramides)
sphingolipids
negative intrinsic curvature
Count
I
[H2O2] (μM)
0
500
PtSer:Annexin V
Apoptosis
PtSer:Annexin V Binding
Time (hr)
J
Annexin V
+H2O2
K
Caspase-9
Cleaved Caspase-3
CTRL +H2O2
ELOVL2KD +H2O2

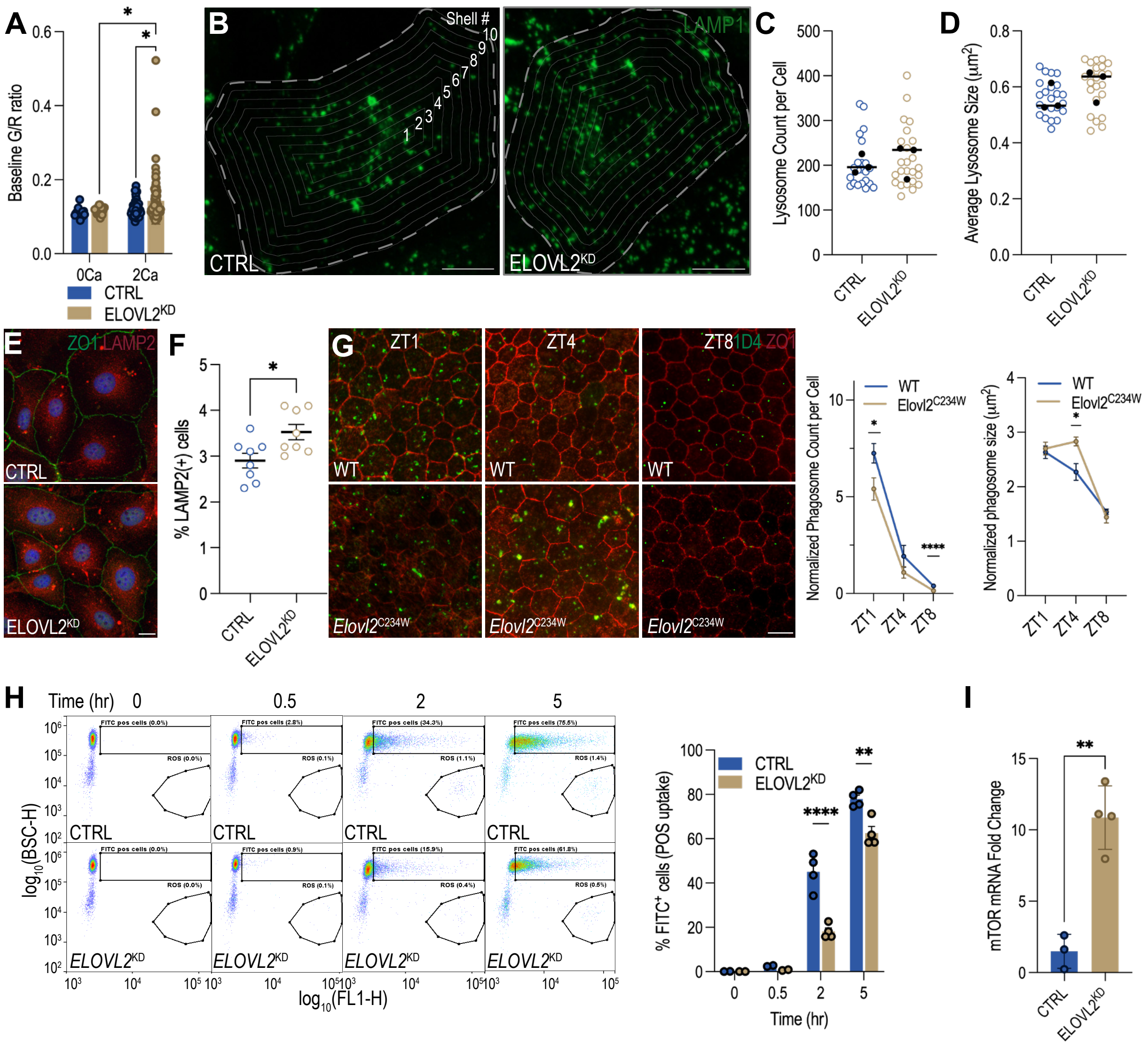

A
Baseline G/R ratio
0Ca
2Ca
CTRL
ELOVL2KD
B
Shell #
CTRL
ELOVL2KD
LAMP1
C
Lysosome Count per Cell
D
Average Lysosome Size (µm2)
E
ZO1 LAMP2
F
% LAMP2(+) cells
G
ZT1
ZT4
ZT8
1D4 ZO1
WT
Elovl2C234W
Normalized Phagosome Count per Cell
Normalized phagosome size (µm2)
H
Time (hr)
log10(BSC-H)
log10(FL1-H)
FITC pos cells
ROS
% FITC+ cells (POS uptake)
I
mTOR mRNA Fold Change

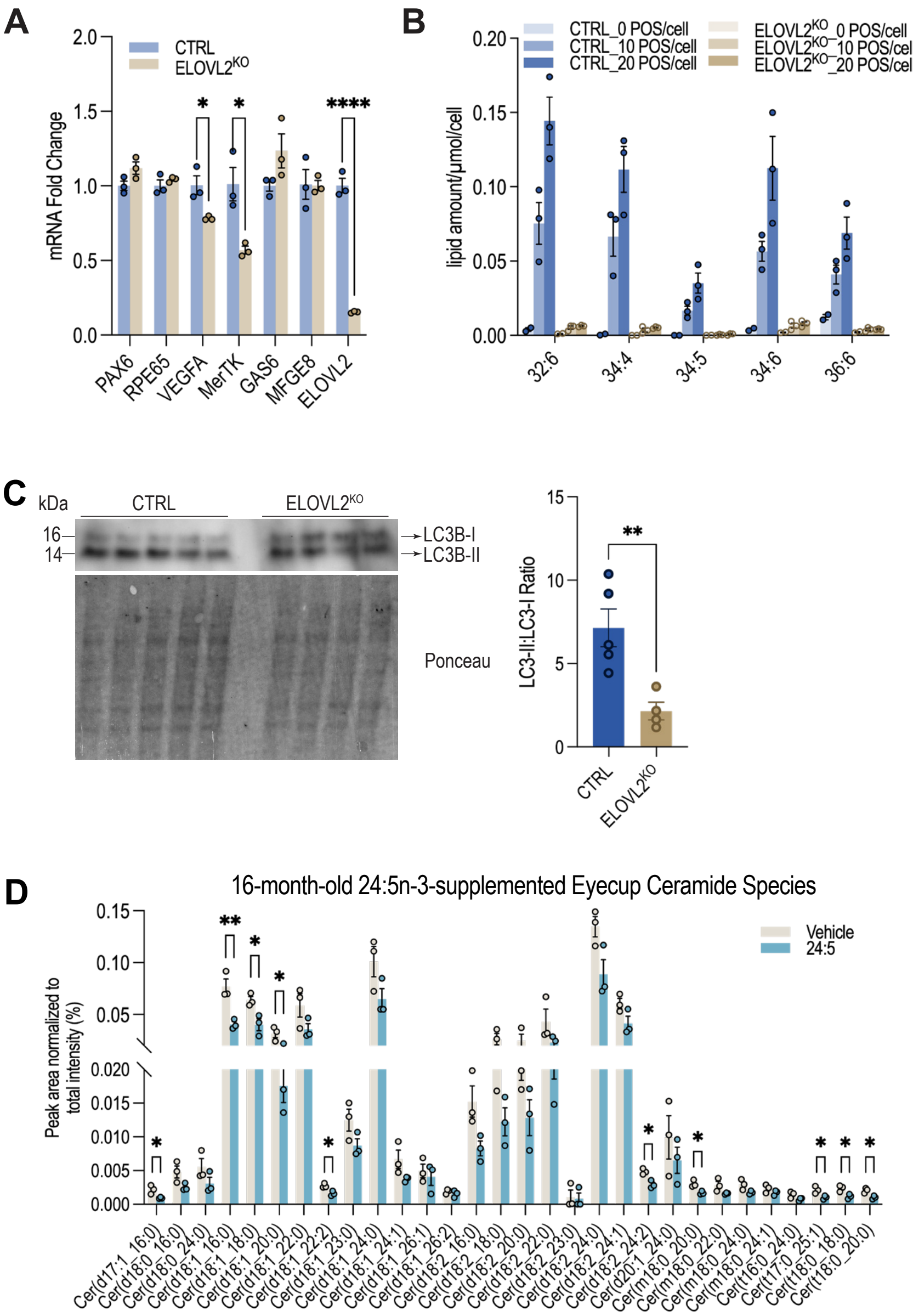
A
mRNA Fold Change
2.0
1.5
1.0
0.5
0.0
CTRL
ELOVL2KO
*
*
****
PAX6
RPE65
VEGFA
MerTK
GAS6
MFGE8
ELOVL2
B
lipid amount/µmol/cell
0.20
0.15
0.10
0.05
0.00
CTRL_0 POS/cell
CTRL_10 POS/cell
CTRL_20 POS/cell
ELOVL2KO_0 POS/cell
ELOVL2KO_10 POS/cell
ELOVL2KO_20 POS/cell
32:6
34:4
34:5
34:6
36:6
C
kDa
CTRL
ELOVL2KO
16
14
LC3B-I
LC3B-II
Ponceau
LC3-II:LC3-I Ratio
15
10
5
0
**
CTRL
ELOVL2KO
D
16-month-old 24:5n-3-supplemented Eyecup Ceramide Species
Peak area normalized to total intensity (%)
0.15
0.10
0.05
0.020
0.015
0.010
0.005
0.000
Vehicle
24:5
**
*
*
*
*
*
*
*
*
*
Cer(d17:1_16:0)
Cer(d18:0_16:0)
Cer(d18:0_24:0)
Cer(d18:1_16:0)
Cer(d18:1_18:0)
Cer(d18:1_20:0)
Cer(d18:1_22:0)
Cer(d18:1_22:2)
Cer(d18:1_23:0)
Cer(d18:1_24:0)
Cer(d18:1_24:1)
Cer(d18:1_26:1)
Cer(d18:1_26:2)
Cer(d18:2_16:0)
Cer(d18:2_18:0)
Cer(d18:2_20:0)
Cer(d18:2_22:0)
Cer(d18:2_23:0)
Cer(d18:2_24:0)
Cer(d18:2_24:1)
Cer(d18:2_24:2)
Cer(d20:1_24:0)
Cer(m18:0_20:0)
Cer(m18:0_22:0)
Cer(m18:0_24:0)
Cer(m18:0_24:1)
Cer(t16:0_24:0)
Cer(t17:0_25:1)
Cer(t18:0_18:0)
Cer(t18:0_20:0)
