# Supplementary material for: Lipid-driven membrane remodeling engages a lysosome-dependent adaptive repair program during retinal aging
Source: Mol Neurodegener. 2026 Aug 29;21:56. doi: 10.1186/s13024-026-00990-w (PMC13563809; doi:10.1186/s13024-026-00990-w)
Supplement: Supplementary file 2 [file 13024_2026_990_MOESM1_ESM.docx]

**Fig. S1. PUFA depletion in *Elovl2*^C234W^ mice correlates with molecular and structural changes in the RPE.**

(A) Detailed presentation of lipid analysis presented in Figure 1C. Relative abundances of LC- and VLC-PUFAs in eyecups from 18-month-old wild-type mice relative to 3-month-old wild-type mice (n = 5 per group).

(B) Representative images of central and peripheral regions from wild-type and *Elovl2*^C234W^ mice. Scale bars, 20 μm.

(C) Representative in vivo two-photon fundus images from wild-type and *Elovl2*^C234W^ mice acquired at 760 nm excitation. Top: grayscale intensity images. Middle: fluorescence lifetime imaging microscopy (FLIM) semicircle phasor plots. Clusters are color-coded from blue to red, with red indicating highest pixel density. Bottom: FLIM phasor images corresponding to the phasor plots.
(D) Representative grayscale two-photon autofluorescence images acquired across 400–660 nm, 400–550 nm, and 580–680 nm wavelength ranges.
Statistical significance was determined using unpaired two-tailed t tests (A). Data are mean ± SEM. *P < 0.05, **P < 0.01, ***P < 0.001, ****P < 0.0001. Lipidomics data from mouse eyecups have been deposited in Dryad (DOI 10.5061/dryad.34tmpg51p) (A).

**Fig. S2. Disruption of LC-PUFA synthesis drives ultrastructural defects and lipid remodeling.**

(A) Relative abundances of LC- and VLC-PUFAs in eyecups from 18-month-old *Elovl2*^C234W^ mice relative to age-matched wild-type mice (n = 3 per group).
(B and C) Heatmap (B) and relative abundances (C) of lipid classes in *Elovl2*^C234W^ eyecups (n = 2 per group).
(D) Relative abundances of total PUFA-esterified lipids and DHA-containing lipids (n = 2 per group).
(E) Relative abundances of lipid classes in eyecups from 15-month-old *Elovl2*^C234W^ mice relative to 18-month-old wild-type mice (n = 3 per group).
(F) Relative abundances of PUFA-esterified and DHA-containing lipids in 15-month-old *Elovl2*^C234W^ mice relative to 18-month-old wild-type mice (n = 3 per group).
(G) Pearson correlation analysis of all detected lipid species (n = 3 per group).
Statistical significance was determined using unpaired two-tailed t tests (A to C and E). Data are mean ± SEM. *P < 0.05, **P < 0.01, ***P < 0.001, ****P < 0.0001. Lipidomics data from mouse eyecups have been deposited in Dryad (DOI 10.5061/dryad.34tmpg51p) (A-G).

**Fig. S3. ELOVL2 loss remodels the plasma membrane lipidome and induces membrane stress.**

(A) qPCR analysis of *RPE65* and *ELOVL2* expression in differentiated control and *ELOVL2*^KD^ cells (n = 4 per group). The dashed line indicates expression in undifferentiated ARPE-19 cells. Data are mean ± SEM.
(B) Relative abundances of LC- and VLC-PUFAs and lipid classes in *ELOVL2*^KD^ cells (n = 4 per group).
(C and D) Heatmap (C) and relative abundances (D) of significantly altered lipid classes in whole-cell lipidomics (n = 4 per group).
(E) Relative abundances of individual ceramide species (n = 4 per group).
(F) Immunoblot analysis of nuclear, cytoplasmic, organelle, and plasma membrane fractions. Fraction purity was assessed using Na⁺/K⁺-ATPase (plasma membrane), α-tubulin (cytoplasm), and TOM20 (organelles).
(G) Heatmap of ceramide saturation profiles (SFA, MUFA, PUFA) in eyecups from 18-month-old versus 3-month-old wild-type mice (n = 5 per group).
(H) LION enrichment analysis of altered lipid species in plasma membrane fractions.
(I) Real-time measurement of phosphatidylserine externalization (Annexin V) and apoptosis activation following treatment with 500 μM H₂O₂ (n = 3 for 0 μM and n = 4 for 500 μM).
(J and K) Representative confocal images of Annexin V (J) and caspase-9/cleaved caspase-3 (K) after H₂O₂ treatment. Annexin V was assessed at 1 hour and caspase activation at 6 hours. Scale bars, 10 μm.
Statistical significance was determined using unpaired two-tailed t tests (A to E and G) or two-way ANOVA (I). Data are mean ± SEM. *P < 0.05, **P < 0.01, ***P < 0.001, ****P < 0.0001. Lipidomics data from *ELOVL2*^KD^ cells have been deposited in Dryad (DOI 10.5061/dryad.34tmpg51p) (B-E, G, H).

**Fig. S4. ELOVL2 deficiency alters lysosomal dynamics and lysosome–plasma membrane fusion.**

(A) Quantification of baseline calcium signaling (minimum 10 GCaMP6f/tdTomato ratios per cell).
(B) Representative images of LAMP1-positive lysosomes with radial segmentation overlays. Scale bars, 10 μm.
(C and D) Quantification of lysosome number (C) and size (D). Data are replicate mean ± SEM (closed circles; n = 3) with individual fields (open circles; >7 per replicate).
(E) Representative images of ZO-1 and LAMP2 staining. Scale bars, 10 μm.
(F) Lysosome–plasma membrane fusion assay. Left: schematic. Right: flow cytometry of surface LAMP2 (n = 8 per group).
(G) Phagosome analysis in RPE flat mounts. Left: representative images (ZT1, ZT4, ZT8). Right: quantification of phagosome number and size (n = 3 animals per group). Scale bars, 20 μm.
(H) POS phagocytosis assay. Uptake (% FITC⁺ cells) measured at indicated time points.
(I) qPCR analysis of *mTOR* expression.
Statistical significance was determined using unpaired two-tailed t tests (C, D, F, I) or two-way ANOVA (A, G, H). Data are mean ± SEM. *P < 0.05, **P < 0.01, ***P < 0.001, ****P < 0.0001.

**Fig. S5. ELOVL2 enzymatic activity is required for lipid homeostasis.**

(A) qPCR analysis of RPE differentiation markers (PAX6, RPE65, VEGFA, MERTK, GAS6, MFGE8) and ELOVL2 expression in control and *ELOVL2*^KO^ iPSC-RPE (n = 3 per group).
(B) Lipidomics-based phagocytosis assay measuring ROS-derived VLC-PUFAs (32:6, 34:4, 34:5, 34:6, and 36:6) following POS incubation with *ELOVL2*^KO^ iPSC-RPE (n = 2 wild type cells and n=3 *ELOVL2*^KO^ iPSC-RPE).
(C) Immunoblot analysis of LC3-I and LC3-II with Ponceau S loading control (left) and quantification of autophagic flux (right) (n = 5 wild type cells and n = 4 *ELOVL2*^KO^ iPSC-RPE).
(D) Relative abundances of ceramide species in eyecups from 24:5n-3–supplemented mice (n = 3 per group).
Statistical significance was determined using unpaired two-tailed t tests (A, C, D). Data are mean ± SEM. *P < 0.05, **P < 0.01, ***P < 0.001, ****P < 0.0001. Lipidomics data from *ELOVL2*^KO^ iPSC-RPE and 24:5n-3-supplemented mouse eyecups have been deposited in Dryad (DOI 10.5061/dryad.34tmpg51p) (B, D).
